# Smart control lipid-based nanocarriers for fine-tuning gut hormone secretion

**DOI:** 10.1126/sciadv.adq9909

**Published:** 2024-12-13

**Authors:** Yining Xu, Cécilia Bohns Michalowski, Jackie Koehler, Tamana Darwish, Nunzio Guccio, Constanza Alcaino, Inês Domingues, Wunan Zhang, Valentina Marotti, Matthias Van Hul, Paola Paone, Melitini Koutsoviti, Ben J. Boyd, Daniel J. Drucker, Patrice D. Cani, Frank Reimann, Fiona M. Gribble, Ana Beloqui

**Affiliations:** ^1^ Louvain Drug Research Institute, Advanced Drug Delivery and Biomaterials, Université catholique de Louvain, 1200 Brussels, Belgium.; ^2^ Department of Pharmacy, Institute of Metabolic Diseases and Pharmacotherapy, West China Hospital, Sichuan University, Chengdu 610041, China.; ^3^ Laboratory of Drug-Targeting and Drug Delivery System of the Education Ministry, Department of Clinical Pharmacy and Pharmacy Administration, West China School of Pharmacy, Sichuan University, Chengdu 610041, China.; ^4^ Institute of Metabolic Science, Addenbrooke’s Hospital, University of Cambridge, Hills Road, Cambridge, CB2 0QQ, UK.; ^5^ Lunenfeld-Tanenbaum Research Institute, Mt. Sinai Hospital, Toronto, ON M5G 1X5, Canada.; ^6^ Department of Medicine, University of Toronto, Toronto, ON M5S 2J7, Canada.; ^7^ Louvain Drug Research Institute, Metabolism and Nutrition Group, Université catholique de Louvain, 1200 Brussels, Belgium.; ^8^ Department of Pharmacy, Faculty of Health and Medical Sciences, University of Copenhagen, Universitetsparken 2, 2100 Copenhagen Ø, Denmark.; ^9^ Novo Nordisk A/S, 2760 Måløv, Denmark.; ^10^ Drug Delivery, Disposition and Dynamics, Monash Institute of Pharmaceutical Sciences, Monash University, Parkville, VIC, Australia.; ^11^ WEL Research Institute, Avenue Pasteur, 6, 1300 Wavre, Belgium.; ^12^ Institute of Experimental and Clinical Research (IREC), Université catholique de Louvain, 1200 Brussels, Belgium.

## Abstract

Modulating the endogenous stores of gastrointestinal hormones is considered a promising strategy to mimic gut endocrine function, improving metabolic dysfunction. Here, we exploit mouse and human knock-in and knockout intestinal organoids and show that agents used as commercial lipid excipients can activate nutrient-sensitive receptors on enteroendocrine cells (EECs) and, when formulated as lipid nanocarriers, can bestow biological effects through the release of GLP-1, GIP, and PYY from K and L cells. Studies in wild-type, dysglycemic, and gut *Gcg* knockout mice demonstrated that the effect exerted by lipid nanocarriers could be modulated by varying the excipients (e.g., nature and quantities), the formulation methodology, and their physiochemical properties (e.g., size and composition). This study demonstrates the therapeutic potential of using nanotechnology to modulate release of multiple endogenous hormones from the enteroendocrine system through a patient-friendly, inexpensive, and noninvasive manner.

## Teaser

Lipid nanocarriers can be used to modulate release of multiple endogenous hormones from the enteroendocrine system.

## INTRODUCTION

Enteroendocrine cells (EECs), the largest endocrine system in the human body, are distributed throughout the gastrointestinal tract (*1*). More than 20 gut hormones are released from different types of EECs and act synergistically to fine-tune various physiological processes in and around the gut. K cells and L cells, the most widely studied EECs, are the targets of antidiabetic therapies because of their secretion of gut peptides [e.g., gastric inhibitory peptide (GIP), glucagon-like peptide-1 (GLP-1), and peptide YY (PYY)] with anti-obesity and/or antidiabetic action. GLP-1 and GIP, incretin hormones, play an important physiological role stimulating glucose-dependent insulin secretion from pancreatic β cells and are responsible for a substantial proportion of postprandial insulin release (*2*). PYY, an anorectic gut hormone, acts as a satiety signal controlling appetite and body weight (*3*). Over the years, gut hormone mimetics, exemplified by GLP-1 receptor agonists and coagonists used to treat type 2 diabetes mellitus (T2DM) and obesity (*4*), have gained increasing attention.

Downstream signaling of multiple gut hormone receptors is activated after bariatric surgery and is a key factor in patients’ metabolic improvement (*5*). The robust postprandial changes in multiple gut peptide responses observed in patients postoperatively suggest that approaches that integrate the physiological effects of multiple gut hormones may mimic the effects of bariatric surgery (*6*, *7*). Ongoing studies have also confirmed that a combination of multiple physiological effects induced by different intestinal peptides is better for patients with obesity or diabetes than the current single gut peptide therapy (*8*, *9*). Newer multiagonist peptides offer combinatorial metabolic efficacy, such as tirzepatide, a dual GIP/GLP-1 agonist (*10*–*12*). Coadministration of agonists and antagonists of gut peptide receptors has also shown some therapeutic promise, such as the coadministration of a GLP-1 agonist and a GIP antagonist (*13*, *14*). However, the majority of current GLP-1–based therapeutics are injected. Achieving modulation of two or more gut hormones in patients through oral therapies targeting different regions of the gut simultaneously remains challenging.

Modulating release of endogenous stores of GLP-1 and other gut hormones has the potential to offer multifaceted beneficial effects similar to those achieved by bariatric surgery on metabolic dysfunction (e.g., control of appetite and glucose homeostasis) (*15*). Dietary interventions and nutrient manipulations (lipids, carbohydrates, proteins, etc.) regulate the endogenous release of gastrointestinal hormones (*15*, *16*). However, nutritional strategies alone cannot meet the clinical needs of most patients with obesity and diabetes. We recently exploited the physiology of one type of EEC (enteroendocrine L cells) in drug delivery, successfully developing a lipid-based nanocarrier to increase the release of endogenous GLP-1 via a noninvasive delivery (oral) route (*17*–*19*). Using this technique, we were able to achieve therapeutically relevant levels of GLP-1 in vivo. Nevertheless, ongoing challenges remain in developing smart control drug delivery systems that can exert multibiological effects by rationally modulating the release of multiple gut peptides from EECs.

EECs are subdivided into different cell types based on their location and main secretory hormones (*20*). K cells are mainly located in the proximal small intestine (duodenum and jejunum), whereas L cells are found in greater numbers in the distal intestine (ileum and colon) (*21*). Strong evidence has also demonstrated that the physiochemical properties of drug delivery systems, such as particle size, may affect the physiological interaction between nanoparticles and targeted areas in gut (*22*). We hypothesized that rationally designed nanocarriers with different physicochemical properties may target different EEC populations located in different segments of the gastrointestinal tract, modulating secretion of different gut hormone secretion for a targeted disease treatment.

Nutrients, such as lipids, can interact with various G protein–coupled receptors (GPCRs) expressed on EECs resulting in modulation of the secretion of different gut hormones (*23*). For example, free fatty acid receptors 1 to 4 are lipid-sensing receptors activated by short- and long-chain fatty acids that regulate the release of GLP-1 and GIP (*24*, *25*). GPR119 is a receptor for lipid derivatives including long-chain monoacylglycerols and participates in postprandial secretion of GIP, GLP-1, and PYY (*26*). Although lipid intake can regulate gut hormone secretion, excessive lipid intake leads to a systemic energy imbalance, causing obesity and an increased risk of T2DM (*27*). Alternatively, selective manipulation of EEC nutrient sensors (e.g., GPCRs) is considered an emerging strategy in the treatment of metabolic diseases (*28*). Lipids acting on these nutrition-sensitive receptors are widely present in a wide range of generally recognized as safe (GRAS) excipients used in the drug delivery field. Thus, exploiting commonly used excipients to activate nutrient receptors on EECs could potentially mimic the enteroendocrine effects of lipid ingestion. In this study, we demonstrate with studies in wild-type (WT), dysglycemic, and gut *Gcg* knockout (KO) mice that, when these excipients are combined and formulated as lipid nanocapsules, they can trigger the secretion of gut hormones. This effect can be modulated by changing the size/composition of the nanocapsules toward the simultaneous secretion of different gut hormones secreted by different EECs located in different regions of the gut. We further encapsulated the GLP-1 analog exenatide (EXE) and conducted an oral glucose tolerance test (OGTT) in obese/diabetic mice to demonstrate that the encapsulation of the peptide would be needed toward a therapeutic effect in the pathological context. Together, this study demonstrates the therapeutic potential of using nanotechnology to modulate release of multiple endogenous hormones from the enteroendocrine system through a patient-friendly, inexpensive, and noninvasive manner.

## RESULTS

### Lipid nanocarriers stimulate gut hormones in murine and human organoids

**Fig. 1.**
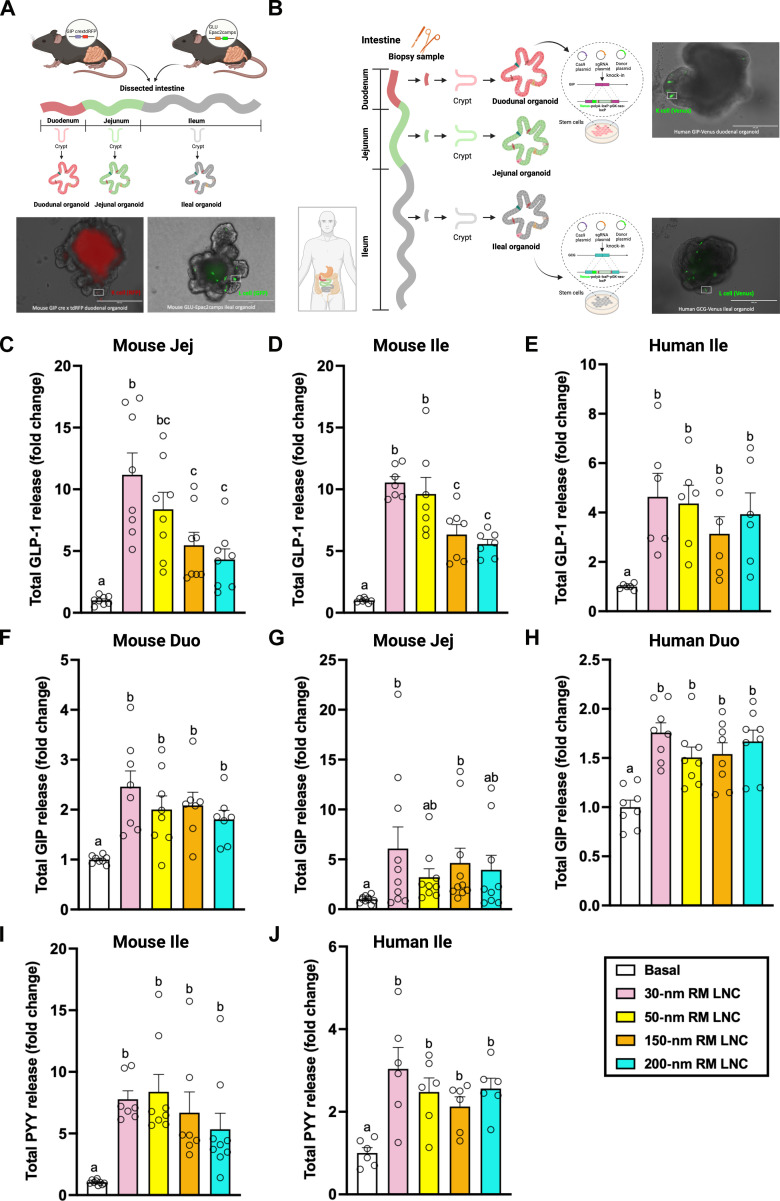
Size effect of lipid nanocarrier–mediated release of antidiabetic hormones in the gut (ex vivo). (**A**) Schematic illustration of the generation of intestinal organoids (duodenal, jejunal, and ileal organoids) from transgenic mice (GIP-cre/tdRFP mice and GLU-EPAC2camp-GFP mice). (**B**) Schematic demonstration of transgenic intestinal organoid (duodenal and ileal) generation from humans. (**C** to **E**) Effects of particle size on GLP-1 secretion mediated by RM LNC in different intestinal segments, including mouse jejunal organoids (C), mouse GLU EPAC2-GFP ileal organoids (D), and human GLU GCG-Venus ileal organoids (E). Extracellular and intracellular GLP-1 levels were determined by enzyme-linked immunosorbent assay (ELISA) after incubation with RM LNC of different sizes (2 mg/ml) on 2D monolayers of intestinal organoids for 2 hours. Total GLP-1 release was calculated using extracellular and intracellular GLP-1 levels. (**F** to **H**) GIP secretion mediated by RM LNC with different particle sizes in different intestinal segments ex vivo, including mouse GIP-cre/tdRFP duodenal organoids (F), mouse jejunal organoids (G), and human GIP-Venus duodenal organoids (H). After being coincubated with nanoparticles (2 mg/ml) at 37°C for 2 hours, total GIP release was calculated by measuring the total GIP levels in the supernatant and the lysate. (**I** and **J**) RM LNC with different sizes (from 30 to 200 nm) stimulated the secretion of PYY from mouse and human ileum ex vivo, including mouse GLU-EPAC2-GFP ileal organoids (I) and human GLU-Venus ileal organoids (J). After the nanoparticles (2 mg/ml) were incubated with 2D monolayers of intestinal organoids for 2 hours, the extracellular and intracellular total PYY levels were determined by ELISA. Total PYY release was calculated from the extracellular and intracellular total PYY levels. For all figures, data are presented as mean ± SEM (*n* = 6 to 10), corresponding to three to five independent experiments. Data with different superscript letters are significantly different (*P* < 0.05), determined by one-way ANOVA followed by Tukey’s post hoc test.

To understand how gut hormone secretion is stimulated by lipid nanocarriers, ex vivo intestinal transgenic organoid models were used (Fig. 1, A and B). GLP-1 secretion from mouse and human ileal organoids with fluorescently labeled L cells, plated in two-dimensional (2D) cultures, was increased by incubation with 200-nm reverse micelle-loaded lipid nanocapsules (RM LNC) (concentrations ranging from 0.5 to 2 mg/ml; fig. S1, A and B), confirming the biological activity of the developed nanocarriers to induce GLP-1 secretion. The morphology of enteroendocrine L cells identified by their green fluorescence showed no obvious cytotoxicity in the 2D monolayers after coincubation with the nanoparticle suspensions at the tested concentrations (figs. S2 and S3). An absence of cytotoxicity of nanoparticles in intestinal organoids was further confirmed by lactate dehydrogenase (LDH) and 3-(4,5-dimethylthiazol-2-yl)-(2,5-diphenyltetrazolium bromide) (MTT) assays (fig. S4).

We investigated the effect of the size/composition ratio of RM LNC ex vivo. RM LNC presenting different particle sizes, including 30, 50, 150, and 200 nm, were developed by adjusting the ratio of same excipients in the formulations (tables S1 and S2). The ability of RM LNC to trigger GLP-1 release was first assessed in murine GLUTag cells in vitro, derived from endocrine carcinoma of the large bowel in transgenic mice expressing large T antigen under the control of the proglucagon promoter. Figure S5 shows that RM LNC presenting a larger particle size were associated with a higher secretion of GLP-1 from GLUTag cells, consistent with our previous findings (*17*). In the intestinal organoid model, RM LNC were highly effective at stimulating GLP-1 release (up to 10-fold above the baseline) (Fig. 1, C to E), but particle size was less important, with a significantly greater effect of small sized particles evident in mouse but not human. The GLP-1 secretory effect was evident in organoids from different segments of the mouse and human intestines, including mouse jejunal organoids (Fig. 1C), mouse ileal organoids (Fig. 1D), and human ileal organoids (Fig. 1E). The observed differences in GLP-1 secretory profiles mediated by lipid-based nanocarriers in intestinal organoids compared with GLUTag cells validates the use of organoid models that are closer in properties to native L cells than the immortalized cell line.

To understand whether particle size could differentially affect the secretion of other intestinal hormones (such as GIP and PYY), we conducted further secretory studies of GIP and PYY in mouse and human intestinal organoids. GIP is secreted from enteroendocrine K cells in the proximal gut (e.g., duodenum and proximal jejunum) (*29*), whereas PYY is released from enteroendocrine L cells in the distal gut (e.g., ileum) (*30*). We found that RM LNC increased GIP release from mouse duodenal organoids, mouse jejunal organoids, and human duodenal organoids (Fig. 1, F to H) and also increased PYY release from mouse and human ileal organoids (Fig. 1, I and J). No dependence on particle size was evident. Complementary data on extracellular and intracellular levels of GLP-1, GIP, and PYY in the organoids at the end of the incubations are shown in figs. S6 to S8.

### Secretory effects of lipid excipients in mouse and human intestinal organoids

The biological effect triggered by the developed nanosystems may be explained by one excipient of the formulation, a combination, or the whole nanocarrier per se. Table S3 shows the concentrations of the main components of RM LNC, namely Solutol HS 15, Labrafac lipophile WL 1349, Peceol, and Lipoid S100, at tested concentrations ex vivo. We tested the contribution of these individual components to the hormone stimulation effect triggered by RM LNC. Oils used in RM LNC preparation are mainly composed of derivatives of fatty acids. Labrafac lipophile WL 1349 consists of triglycerides of caprylic and capric acids [medium-chain fatty acids (MCFAs); C8 and C10], and Peceol consists of mono-, di-, and triglycerides of oleic acid [long-chain fatty acids (LCFA; C18)]. Both oils significantly enhanced GLP-1 release in intestinal organoids (fig. S9A). Peceol even at the lowest test concentration showed a better GLP-1 secretory effect when compared with Labrafac lipophile WL 1349 at higher concentrations, suggesting that LCFA may be stronger stimuli of GLP-1 release than MCFAs. Solutol HS 15 (polyoxyethylene esters of 12-hydroxystearic acid) significantly increased GLP-1 secretion in mouse ileal organoids (fig. S9A), which was not reproduced by its constituent LCFA 12-hydroxystearic acid (fig. S9C and table S4). Another surfactant, Lipoid S100 containing soybean phosphatidylcholine, failed to increase GLP-1 secretion (fig. S9B), possibly due to the low concentrations of Lipoid S100 in the formulations at the tested particle concentration. We also found that mixtures of Labrafac lipophile WL 1349, Peceol, and Solutol HS 15 exerted a similar biological effect compared with the nanocarriers (fig. S9A). These data suggest that excipients containing such lipids can act as active components of nanocarriers, mimicking nutrient ligands in the gut to trigger a biological effect by the carriers per se. Following the size particle–dependent and lipid excipient dose-dependent behaviors on RM LNC–inducing GLP-1 secretion in the organoids, we chose the 30- and 200-nm RM LNC to conduct the following ex vivo/in vivo studies.

### Role of nutrient sensors in the secretory effect of lipid nanocarriers

**Fig. 2.**
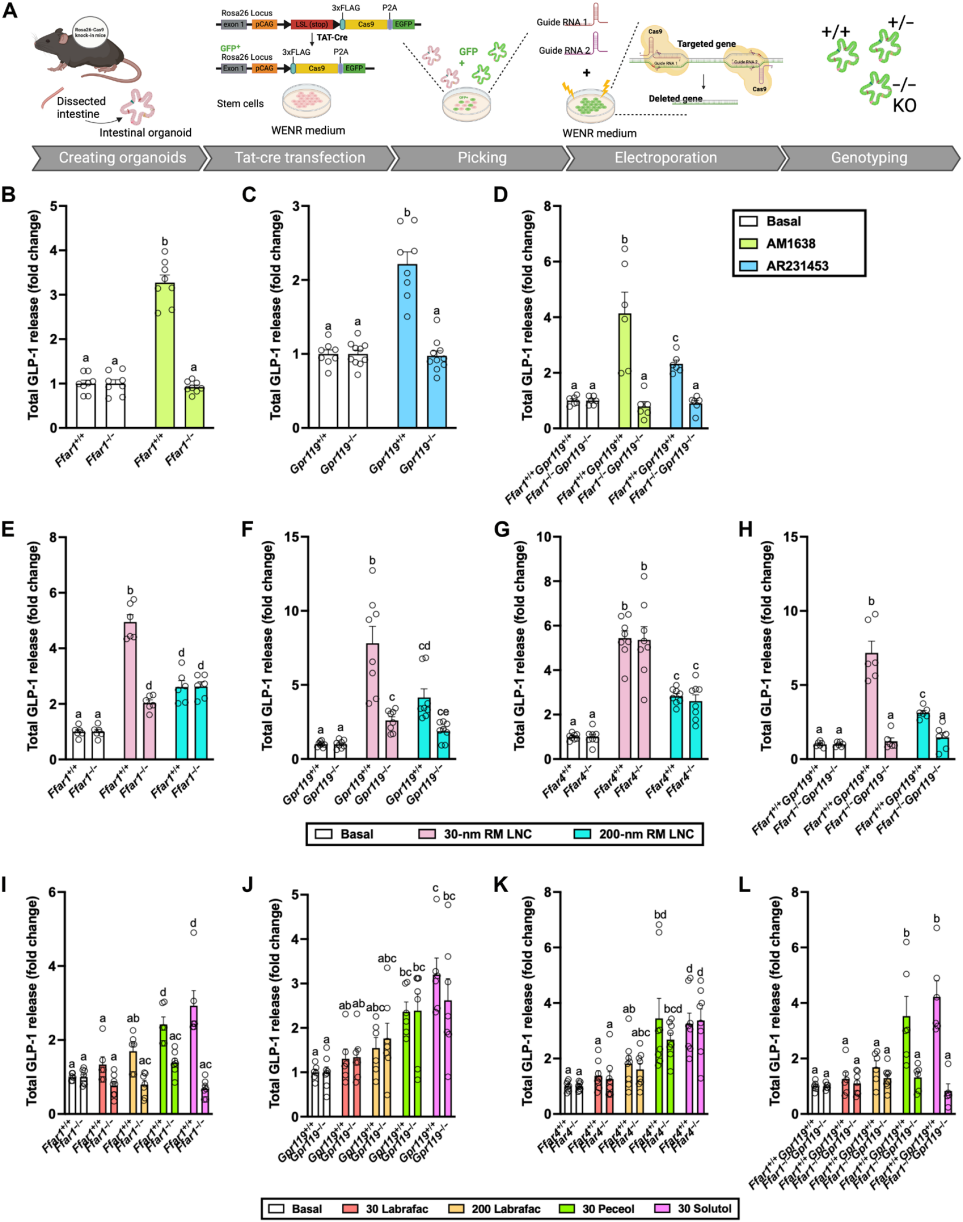
Generation of mouse GPCR KO ileal organoids and role of lipid excipients in the biological effect triggered by lipid-based nanocarriers. (**A**) Schematic illustration of the creation of mouse ileal KO organoids. (**B** to **D**) Validation of different GPCR KO mouse ileal organoids by using different agonists. GLP-1 secretion from 2D monolayers of cas9-GFP mouse ileal *Ffar1*^+/+^ and *Ffar1*^−/−^ organoids (B), cas9-GFP mouse ileal *Gpr119*^+/+^ and *Gpr119*^−/−^ organoids (C), and cas9-GFP mouse ileal *Ffar1*^+/+^*Gpr119*^+/+^ and *Ffar* ^−/−^*Gpr119*^−/−^ organoids (D) after incubation with the FFAR1 agonist AM1638 (10 μM) or the GPR119 agonist AR231453 (0.1 μM) for 2 hours. The data are presented as mean ± SEM (*n* = 6 to 10), corresponding to three to five independent experiments. (**E** to **H**) GLP-1 secretion from cas9-GFP mouse ileal WT (^+/+^) organoids and *Ffar1*^−/−^ (E), *Gpr119*^−/−^ (F), *Ffar4*^−/−^ (G), and *Ffar1*^−/−^*Gpr119*^−/−^ (H) organoids after incubation with 30- and 200-nm RM LNC for 2 hours. The data are presented as mean ± SEM (*n* = 6 to 8), from three to four experiments. (**I** to **L**) Effects of the main components or RM LNC on GLP-1 secretion from WT and different GPCR KO organoids. GLP-1 secretion from 2D monolayers of mouse cas9-GFP mouse WT (^+/+^) organoids and *Ffar1*^−/−^ (I), *Gpr119*^−/−^ (J), *Ffar4*^−/−^ (K), and *Ffar1*^−/−^*Gpr119*^−/−^ (L) organoids, on addition of 0.651 μM Labrafac lipophile WL 1349 (30 Labrafac), 3.598 μM Labrafac lipophile WL 1349 (200 Labrafac), 0.666 μM Peceol (30 Peceol), and 0.981 μM Solutol HS 15 (30 Solutol) in the presence of basal buffer (mean ± SEM, *n* = 5 to 8 from three to four experiments). For all figures, total GLP-1 release was calculated from the total GLP-1 levels in the supernatant and the lysate. Data with different superscript letters are significantly different (*P* < 0.05), determined by one-way ANOVA followed by Tukey’s post hoc test.

The LCFA receptors GPR40 [free fatty acid receptor 1; (FFAR1)] and GPR120 (FFAR4) (*31*) and the monoacylglycerol receptor GPR119 were considered as potential mediators of the stimulatory effect of RM LNC and have been linked to stimulation of gut hormone secretion (*26*, *32*, *33*). To examine the potential contribution of Gq signaling, the downstream pathway from FFAR1 and FFAR4, we monitored GLP-1 release induced by RM LNC and their individual excipient components (30 and 200 nm) in mouse ileal organoids using the Gq inhibitor UBO-QIC (FR900359) (*34*). UBO-QIC impaired GLP-1 secretion induced by RM LNC, Solutol HS 15, or Peceol (fig. S10), suggesting the involvement of Gq-coupled receptors, whereas the response to Labrafac was unaffected. To delineate the GPCRs involved in lipid nanocarrier–induced hormone secretion, we next used the CRISPR-Cas9 technique to generate KO mouse ileal organoid models, including FFAR1 KO (*Ffar1*^−/−^), GPR119 KO (*Gpr119*^−/−^), and FFAR4 KO (*Ffar4*^−/−^) (Fig. 2A). Using pairs of single guide RNAs (sgRNAs) (table S5), Cas9-mediated double-strand break resulted in the generation of targeted gene WT (+/+), heterozygous (+/−), and homozygous (KO, −/−) lines for each gene, as confirmed by polymerase chain reaction (PCR) screening (table S6 and figs. S11 to S13). We used the FFAR1 agonist AM1638 and the GPR119 agonist AR231453 to confirm the functional loss of FFAR1 (Fig. 2B) and GPR119 (Fig. 2C) in these KO models. Figure 2 (E to G) shows that FFAR1 and GPR119 are involved in RM LNC–mediated GLP-1 secretion, whereas FFAR4 had no clear contribution. To extend these observations, we also generated FFAR1 and Gpr119 double gene KO mouse ileal organoids (*Ffar1*^−/−^*Gpr*119^−/−^) using the same technology. Genotyping results (fig. S14) and a loss of stimulation by FFAR1 and GPR119 agonists (Fig. 2D) confirmed the successful generation of mouse ileal *Ffar1*^−/−^*Gpr*119^−/−^organoids. We found that the stimulatory effects of both 30- and 200-nm RM LNC on GLP-1 secretion were completely suppressed in *Ffar1*^−/−^*Gpr*119^−/−^ mouse ileal organoids (Fig. 2H). In addition, we investigated whether the loss of these receptors affected GLP-1 secretion induced by the individual components of the RM LNC. As shown in Fig. 2 (I to L), we observed that FFAR1, but not GPR119 or FFAR4, was involved in responses to Labrafac, Peceol, and Solutol HS. The GPR119 dependence of the response to RM LNC might be attributable to the small amount of Lipoid S100 (phosphatidylcholine) contained in the formulation, although the concentration of Lipoid S100 used in RM LNC per se was not able to increase GLP-1 release on its own. Complementary data on extracellular and intracellular GLP-1 levels measured in Cas9-GFP mouse ileal WT and KO organoids are supplied in figs. S15 to S17.

**Fig. 3.**
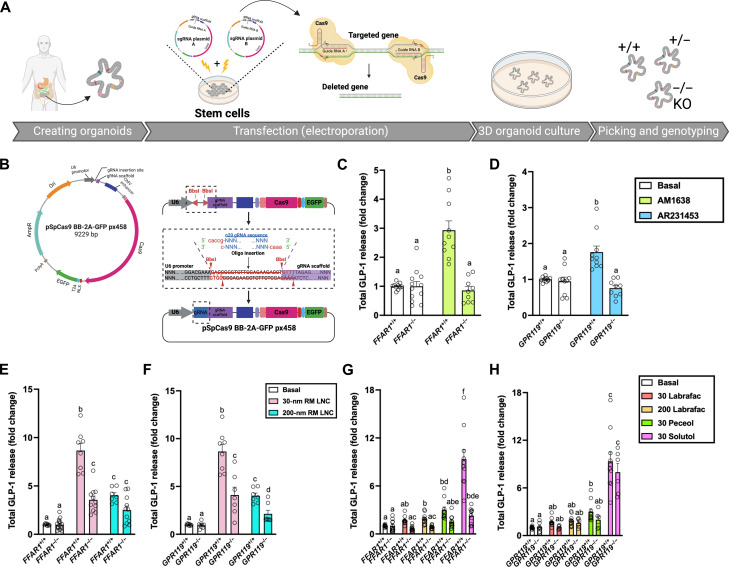
Generation of human GPCR KO ileal organoids and role of lipid excipients in the biological effect triggered by lipid nanocarriers. (**A**) Schematic of the transfection and KO generation strategy for human ileal organoids using CRISPR-Cas9. Human ileal organoids were transfected with two sgRNA plasmids. Single clonal KO organoids were picked and used to establish clonal lines. (**B**) Cloning strategy for px458-based sgRNA plasmid. Each guide is cloned into a separate pX458 plasmid using *BbsI* Golden Gate cloning. Briefly, 20 nucleotides corresponding to the recognition sequences are synthesized as oligonucleotides with *BbsI*-compatible ends and inserted between the U6 promoter and the scaffold RNA (short hairpin RNA) after *BbsI* digestion in plasmids, forming sgRNA plasmid A or sgRNA plasmid B. (**C** and **D**) Validation of human ileal FFAR1 KO organoids (C) and human ileal GPR119 KO organoids (D). GLP-1 secretion from 2D monolayers of human GLU-Venus ileal *FFAR1*^+/+^ and *FFAR1*^−/−^ organoids (C) and human GLU-Venus ileal *GPR119^+/+^* and *GPR119^−/−^* organoids (D) after incubation with the FFAR1 agonist AM1638 (10 μM) or the GPR119 agonist AR231453 (0.1 μM) for 2 hours (mean ± SEM; *N* = 4 to 5, *n* = 8 to 12). (**E** and **F**) GLP-1 secretion from human GLU-Venus ileal WT (^+/+^) organoids and *FFAR1*^−/−^ (E) and *GPR119*^−/−^ (F) organoids after incubation with RM LNC for 2 hours (mean ± SEM; *N* = 3 to 5, *n* = 7 to 15). (**G** and **H**) GLP-1 secretion from 2D monolayers of human GLU-Venus ileal WT organoids and FFAR1 KO (G) and GPR119 KO (H) organoids, on addition of 0.651 μM Labrafac lipophile WL 1349 (30 Labrafac), 3.598 μM Labrafac lipophile WL 1349 (200 Labrafac), 0.666 μM Peceol (30 Peceol), and 0.981 μM Solutol HS 15 (30 Solutol) in the presence of basal buffer (mean ± SEM; *N* = 3 to 5, *n* = 6 to 12). Data with different superscript letters are significantly different (*P* < 0.05), determined by one-way ANOVA followed by Tukey’s post hoc test. CMV, cytomegalovirus.

As we found that FFAR1 and GPR119 to be the most important receptors in RM LNC–mediated GLP-1 secretion in mouse intestinal organoids (Fig. 2), we proceeded to assess the translational applicability of these findings with human organoids. We generated GLU-Venus human ileal *FFAR1*^−/−^ and *GPR119*^−/−^ organoids by CRISPR-Cas9 technology using a pair of sgRNA (table S7) plasmids to induce two double-strand breaks on the targeted genome (Fig. 3, A and B, and figs. S18 and S19, A and B). PCR genotyping (figs. S18C and S19C and table S8) and loss of responsiveness to selective agonists (Fig. 3, C and D) confirmed the successful generation of the KO models.

Consistent with the secretion data from mouse ileal WT and KO organoids, RM LNC–mediated GLP-1 release in human ileal organoids was significantly impaired by KO of either FFAR1 or GPR119 (Fig. 3, E and F). We also quantified GLP-1 release triggered by individual RM LNC components in the KO models. In human intestinal organoids, GLP-1 secretion triggered by Labrafac, Peceol, and Solutol HS was impaired by loss of FFAR1, but not GPR119 (Fig. 3, G and H). Complementary data on the extracellular and intracellular GLP-1 levels measured in GLU-Venus human WT and KO organoids are supplied in figs. S20 to S22. Together, these findings in mouse and human intestinal organoids indicated that the stimulatory effect of RM LNC is achieved via the activation of FFAR1 and GPR119.

### Secretory effect of lipid excipients and fatty acids in vivo

**Fig. 4.**
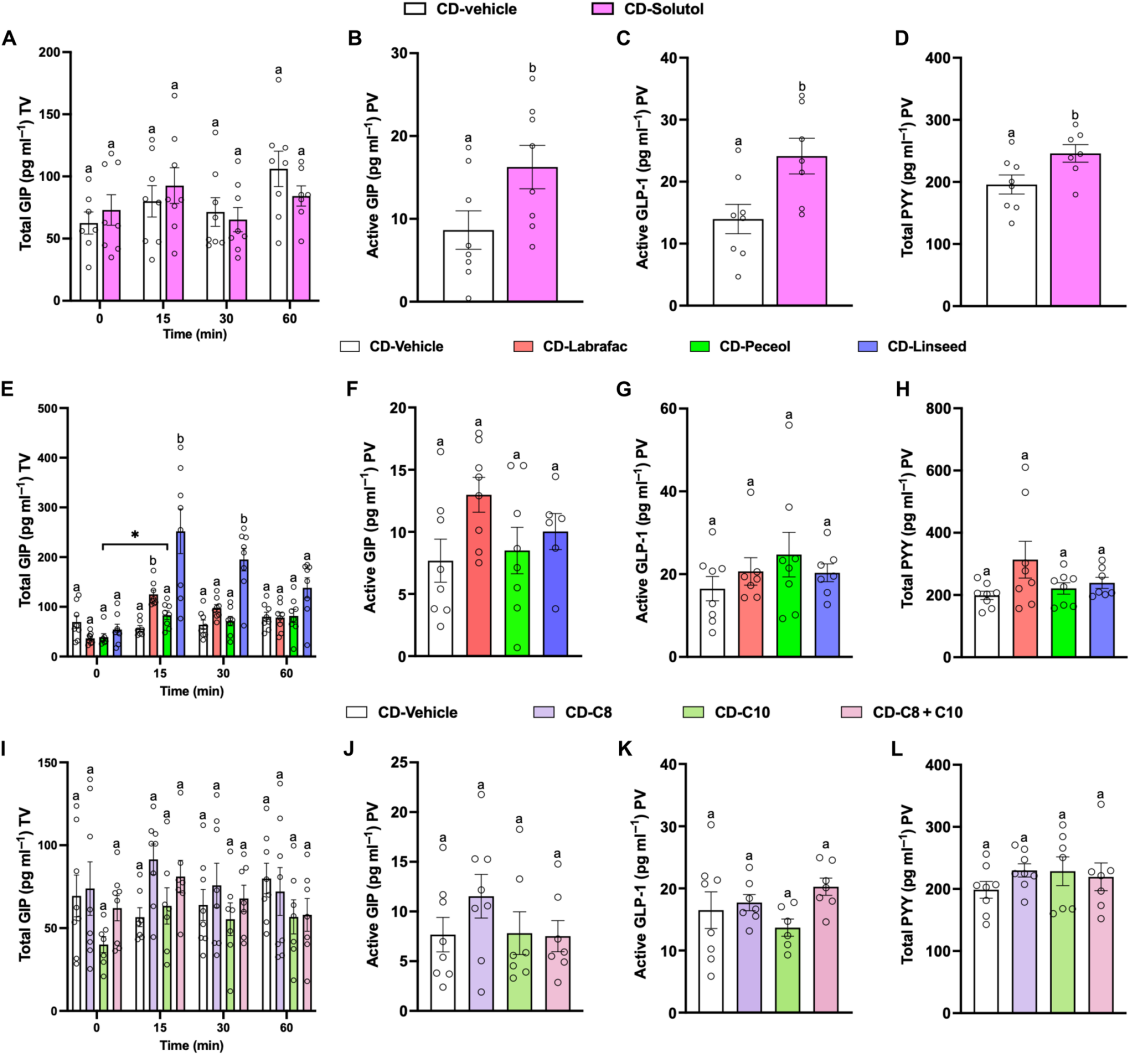
Secretory effect of excipients and fatty acids in vivo. (**A** to **D**) Solutol-mediated gut hormone secretion in mice fed with chow diet (CD). Total GIP levels (A) were measured in mouse plasma from the tail vein (TV) before (0 min) and after 15, 30, and 60 min following oral administration of Milli-Q water (vehicle) and Solutol (0.83 mM/kg, corresponding to that of highest amount used in RM LNC). Plasma active GIP (B), active GLP-1 (C), and PYY levels (D) were measured from the portal vein (PV) 65 min after oral gavage (mean ± SEM; *n* = 6 to 8). (**E** to **H**) Effect of lipid excipients on biological effects triggered by lipid-based nanocarriers in vivo. Plasma total GIP levels (E) were tested from the TV before (0 min) and 15, 30, and 60 min after oral administration of vehicle (dimethyl sulfoxide:Tween 80:Milli-Q water in a 1:1:19 ratio), Labrafac lipophile WL 1349 (4.32 mM/kg), Peceol (0.56 mM/kg), and linseed oil (4.44 mM/kg) (lipid doses corresponding to those of the highest amounts used in the nanocarriers). Plasma active GIP levels (F), active GLP-1 (G), and PYY levels (H) were measured from the PV 65 min after oral gavage (mean ± SEM; *n* = 6 to 8). (**I** to **L**) Free fatty acids in regulating secretion of intestinal hormones in vivo. Plasma total GIP levels (I) were tested from the TV before (0 min) and 15, 30, and 60 min after oral administration of vehicle, caprylic acid (C8; 2.16 mM/kg), capric acid (C10; 2.16 mM/kg), and their mixtures (C8 + C10). Plasma active GIP levels (J), active GLP-1 levels (K), and PYY levels (L) were measured from the PV 65 min post-administration (mean ± SEM; *n* = 6 to 8). Significance was determined by one-way ANOVA followed by Tukey’s post hoc test or Mann-Whitney test. Data with different superscript letters are significantly different (*P* < 0.05).

To further assess the secretory effect of individual RM LNC components in vivo, we measured total GIP and total GLP-1 in the tail vein and active GIP, active GLP-1, and total PYY levels in the portal vein of mice at different time points after oral gavage of the different excipients present in our formulations, including Solutol HS 15, Labrafac lipophile WL 1349, and Peceol. Solutol HS 15 (a surfactant mainly consisting of polyoxyethylene esters of 12-hydroxystearic acid) did not affect tail vein total GIP levels in vivo (Fig. 4A) but increased active GIP, GLP-1, and PYY in the portal vein (Fig. 4, B to D) as well as the total GLP-1 in tail vein blood (fig. S24A), consistent with the stimulation of GLP-1 secretion in the organoid studies ex vivo. Labrafac lipophile WL 1349 (medium-chain triglyceride fatty acids) and Peceol (long-chain mono-, di-, and triglyceride fatty acids) are the two main lipid sources used in our formulation. Peceol administration did not elevate plasma GIP, GLP-1, or PYY levels, and Labrafac lipophile WL 1349 enhanced tail vein GIP levels only at an early time point (15 min after oral administration) (Fig. 4, E to H, and fig. S24B). Excipients used in drug carriers are interchangeable with similar types of components. Linseed oil, a triglyceride of long-chain fatty acids, was used to replace Labrafac lipophile WL 1349 as an oil phase in the formulation, thus replacing MCFAs with LCFAs. Oral gavage of linseed oil resulted in tail vein plasma total GIP levels that were threefold higher than in vehicle controls at 15 min (Fig. 4E) and were stronger and more sustained than in mice given Labrafac lipophile WL 1349, highlighting the important role of LCFAs at increasing GIP secretion after oral administration (Fig. 4E). At these doses, neither GLP-1 levels nor PYY levels were altered, regardless of whether the lipids contained medium-chain triglyceride fatty acids, mixtures of long-chain mono-, di-, and triglyceride fatty acids, or long-chain triglyceride fatty acids (Fig. 4, G and H, and fig. S24C).

The lipid excipients used in our formulation (Labrafac lipophile WL 1349 and Peceol) mainly contain triglycerides of caprylic acid (C8), capric acid (C10), or oleic acid (C18). Peceol, a long-chain (C18) triglyceride, did not affect gut hormone secretion when administered orally at a dose matching the highest quantity contained in our nanoparticles (Fig. 4, E to H). Plasma levels of total GIP, active GIP, active GLP-1, and total PYY were also measured in mice orally administered with caprylic acid (C8), capric acid (C10), or their mixture (C8 + C10), revealing no change in hormone levels in any of the free fatty acid–administered groups compared with vehicle controls (Fig. 4, I to L, and fig. S25B) and suggesting that the concentrations of C8 and C10 corresponding to those in the Labrafac could not per se stimulate the release of intestinal hormones. In summary, these data illustrate that the lipid excipients in RM LNC can exert regulatory effects on different intestinal hormones in vivo and that the profile of gut hormone changes can be varied by replacement of lipids containing different length fatty acids, suggesting their important role as functional components in nanocarriers.

### Biological effect of empty lipid-based nanocarriers and excipient mixtures in vivo

**Fig. 5.**
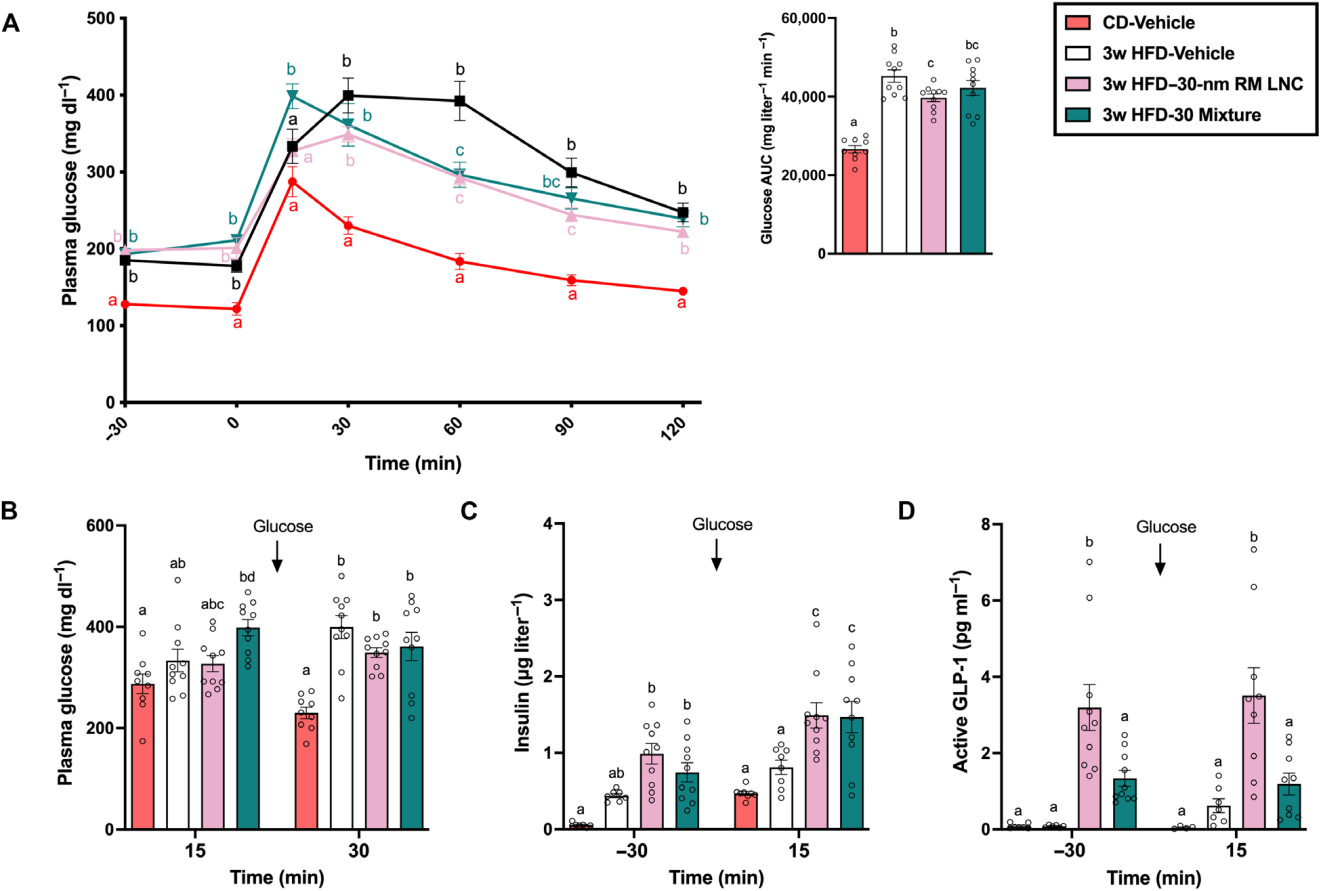
Empty lipid nanocarriers’ and excipient mixtures’ biological effect in glucose metabolism. (**A**) Plasma glucose levels (mg dl^−1^) measured 30 min before and for 120 after oral glucose loading (2 g/kg) and mean glucose AUC (mg dl^−1^ min^−1^) throughout the glucose challenge (*n* = 9-10) after feeding mice a high fat diet (HFD) for with 3 weeks (3w). (**B**) Plasma glucose levels (mg dl^−1^) measured at 15 and 30 min after glucose challenge (*n* = 9 to 10). (**C**) Plasma insulin levels (μg l^−1^) at 30 min before and 15 min after glucose challenge (*n* = 7 to 10). (**D**) Biological effect induced by 30-nm lipid-based nanocarriers (30-nm RM LNC) or mixtures that contained the same amounts of used excipients in 30-nm lipid-based nanocarriers under pathological context (30 Mixture). Plasma active GLP-1 levels (*n* = 5 to 10) quantified 30 min before and 15 min after glucose loading. Empty 30-nm lipid-based nanocarriers (1.62 mg/g lipid dose), 30 Mixture (1.62 mg/g lipid dose), or equivalent volume of water (vehicle) were orally administered 60 min before the oral glucose challenge. Data shown are mean, and data with different superscript letters are significantly different (*P* < 0.05) according to one-way or two-way ANOVA followed by Tukey’s post hoc test.

We found that mixtures of the main functional excipients within our formulations exerted a similar GLP-1 secretory effect compared with the nanocarriers in mouse intestinal organoids (fig. S9A). To further assess their biological effect in vivo, we evaluated the effect of an empty 30-nm lipid-based nanocarrier and the corresponding excipient mixture (corresponding to the same amounts of used excipients in 30-nm lipid formulation) on GLP-1 secretion and glucose metabolism in a high-fat diet (HFD)–induced obese/diabetic model. Mice received a single oral administration of empty 30-nm RM LNC (1.62 mg/g lipid dose), empty 30 Mixture (1.62 mg/g lipid dose), or an equivalent volume of water 60 min before an OGTT in HFD-fed or chow diet (CD)–fed mice (Fig. 5). Oral glucose gavage (2 g/kg) was set as 0 time point. The mixture group was unable to reduce the plasma glucose profile and glucose area under the curve (AUC). Moreover, the mixture group also exhibited higher glucose levels at 15 min compared to the other groups, including the vehicle-treated HFD group. One plausible explanation for this effect is that the mixture contains a high proportion of free surfactants (emulsifying agents). Emulsifying agents have been shown to impair blood glucose control and promote metabolic syndrome (*35*). The glucose AUC of the lipid-based nanocarrier-treated group was significantly lower than that of the vehicle-treated HFD group, but there was no significant difference compared with the mixture group (Fig. 5A). The glucose level of the lipid-based nanocarrier treatment group was significantly lower than that of the mixture group 15 min after oral glucose challenge (Fig. 5B). This better therapeutic effect exerted by a lipid-based nanocarrier on glucose metabolism is consistent to significantly higher active GLP-1 levels 30 min before and 15 min after glucose administration compared to the mixture group (Fig. 5D). Both treated groups could significantly elevate insulin release (Fig. 5C). These data suggest that although functional lipid excipients can exert biological effects on different gut peptides in vivo (Fig. 4, A to H), their simple mixtures (i.e., nondrug delivery systems) are far less effective at regulating intestinal peptide release than through reasonably constructed lipid-based nanocarriers.

### Fine-tuning the secretion of gut hormones via lipid nanocarriers in vivo

Dipeptidyl peptidase IV (DPPIV), an important enzyme in glucose metabolism, cleaves and inactivates GLP-1 and GIP, terminating their ability to stimulate glucose-dependent insulin secretion (*36*). As 200-nm lipid nanocarriers increased endogenous GLP-1 levels in mice (*18*, *19*), we analyzed DPP4 activity in mouse plasma after oral administration of 200-nm RM LNC to investigate whether increased systemic GLP-1 levels are associated with decreased DPP4 activity. Plasma DPP4 activity in the formulation-treated group was not significantly different from the control group (fig. S23). Thus, the elevated GLP-1 levels were not associated with changes in DPP4 activity throughout the body.

**Fig. 6.**
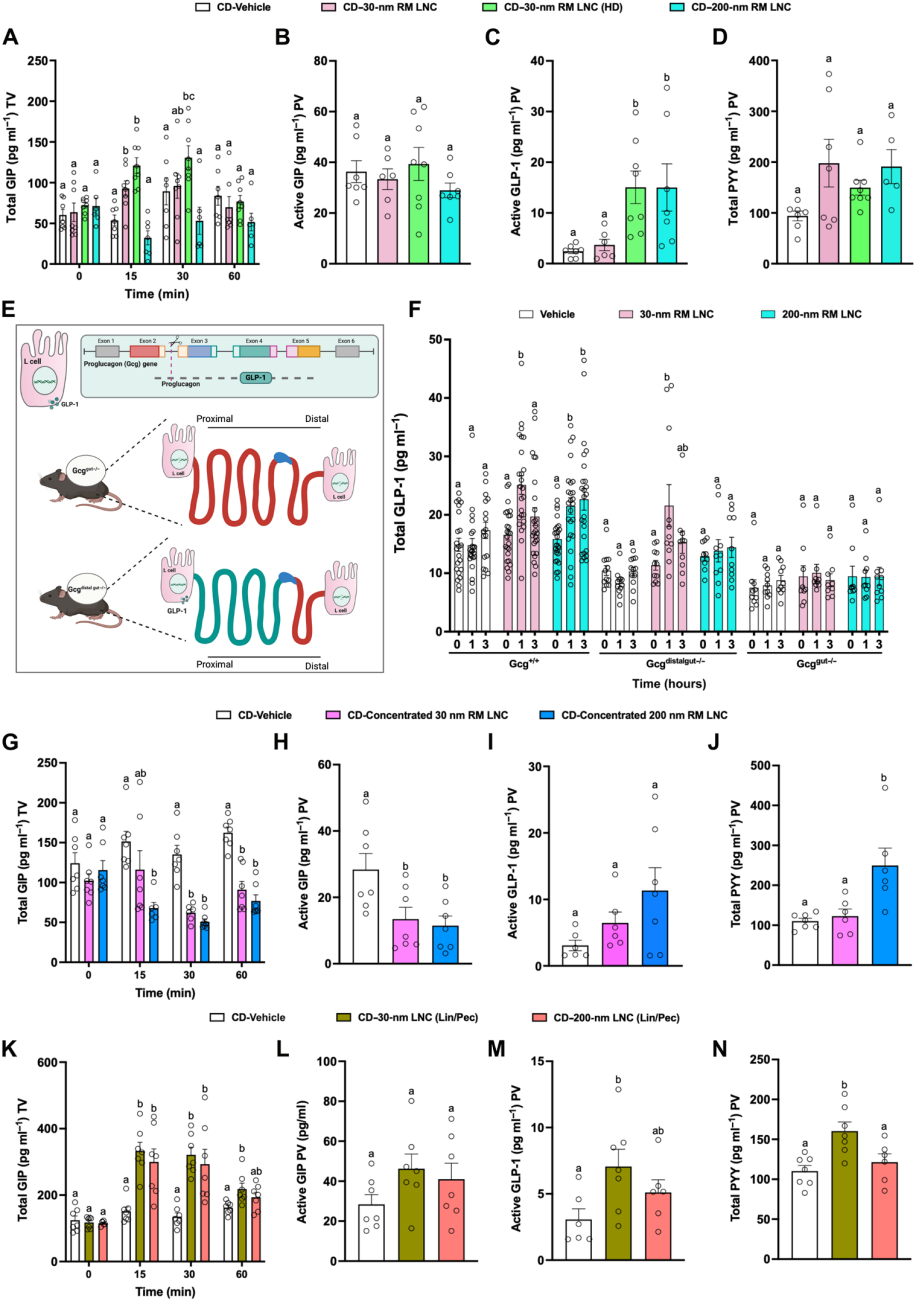
Fine-tuning the stimulation of multiple intestinal hormones through oral administration of lipid-based nanocarriers in mice. (**A** to **D**) Role of size and administration dose on nanocarrier-mediated gut hormone secretion. Plasma total GIP levels (A) were measured from the tail vein (TV) after oral administration of water (vehicle), 30- or 200-nm RM LNC (1.62 mg/g lipid dose), and 30-nm RM LNC with a high dose (2.43 mg/g lipid dose). Plasma active GIP (B), active GLP-1 (C), and PYY (D) levels were measured from the portal vein (PV) 65 min after oral gavage (mean ± SEM; *n* = 6 to 8). (**E**) Schematic graphs of GLP-1–producing gene (*Gcg* gene), mice with elimination of *Gcg* expression in the entire gut (*Gcg*^gut−/−^), and mice with selective elimination of *Gcg* expression in distal gut (*Gcg*^distalgut−/−^). (**F**) Plasma total GLP-1 levels were measured from the TV after oral gavage of Milli-Q water, 30-, and 200-nm RM LNC (1.62 mg/g lipid dose) (mean ± SEM; *n* = 9 to 26). (**G** to **J**) Effect of a concentrated preparation of nanocarriers on gut hormone secretion. Plasma total GIP levels (G) were tested from the TV after oral administration of Milli-Q water, concentrated 30-nm, and 200-nm RM LNC (4.0 mg/g lipid dose). Plasma active GIP (H), active GLP-1 (I), and PYY (J) levels were measured from the PV at 65 min after oral administration (mean ± SEM; *n* = 6 to 7). (**K** to **N**) Effect of excipient type on intestinal hormone responses. Plasma total GIP levels (K) were measured from the TV after oral gavage of Milli-Q water or linseed oil–containing nanocarriers, including 30- and 200-nm RM LNC (Lin/Pec) (1.62 mg/g lipid dose). Plasma active GIP (L), active GLP-1 (M), and PYY (N) levels were measured from the PV 65 min after oral gavage (mean ± SEM; *n* = 6 to 7). Data with different superscript letters are significantly different (*P* < 0.05) according to one-way ANOVA followed by Tukey’s post hoc test.

RM LNC with different sizes produced by different proportions of the same lipid excipients (tables S1 and S2) differentially influenced plasma levels of GLP-1 and GIP (figs. S24 and S25). Specifically, oral administration of both large- and small-size RM LNC (30 and 200 nm) significantly increased the levels of GLP-1 in vivo (fig. S24A). By contrast, whereas 30-nm RM LNC significantly increased endogenous GIP release, 150- and 200-nm RM LNC significantly decreased plasma GIP levels 60 min post-oral administration (fig. S25B). It should be noted that to change the particle size of lipid nanocapsules, we need to adjust the ratio of different components (oils and surfactants) to maintain their structure and morphology. Therefore, both parameters contribute to changes in GIP levels. Both GIP agonists and GIP antagonists represent promising treatments in the field of metabolic disorders (*37*, *38*). Our results raise the possibility that lipid-based nanocarriers, exemplified by RM LNC, can be designed to rationally regulate the release of endogenous GIP by tuning their physiochemical properties (e.g., particle size and component ratio), as an alternative strategy to GIP-based therapies in the pipeline. To further investigate the role of RM LNC in regulating the release of gut hormones in vivo, we tested the effect of oral dosing on plasma GIP, GLP-1, and PYY in the tail and portal veins (Fig. 6, A to D). Elevated GIP levels were observed at early time points (15 and 30 min) following gavage of 30-nm RM LNC, particularly at the higher dose (Fig. 6A), whereas 200-nm RM LNC did not induce GIP release (Fig. 6A). Although total GLP-1 levels in the tail vein were significantly increased in mice treated with 30- and 200-nm RM LNC at a lipid dose of 1.62 mg/g, 60 min post-oral administration (fig. S24A), active GLP-1 in the portal vein differed at the same time point, with a significant elevation observed in the group treated with 200-nm RM LNC but not those given 30-nm RM LNC (Fig. 6C). Nevertheless, 30-nm RM LNC significantly increased levels of active GLP-1 when administered at a higher dose (2.43 mg/g) (Fig. 6C). Portal vein PYY levels showed a trend toward elevation in RM LNC–treated groups but did not reach statistical significance by one-way analysis of variance (ANOVA) (Fig. 6D). Notably, oral administration of individual lipid excipients did not regulate the secretion of multiple intestinal hormones, and their secretory ability was limited (Fig. 4), thus assembling lipids within a delivery system could not only optimize the secretory effect but also achieve the regulation of multiple intestinal hormones after oral administration.

An important feature of gut hormones is that they are secreted by EECs located at different regions of the gut. We hypothesized that the particle size/excipient ratio of lipid-based nanocarriers could drive them to interact with EECs located in different intestinal areas, thereby triggering a different secretory pattern. Gut GLP-1 is mainly produced by enteroendocrine L cells and is encoded by the proglucagon (*Gcg*) gene (Fig. 6E) (*39*). L cells are distributed throughout most of the epithelium of the gut, with the distal gut being the region that harbors the highest density of L cells (*40*). To study the origin/location of RM LNC–mediated GLP-1, we conducted GLP-1 secretory studies in mice harboring a selective KO of Gcg in either the entire mouse gut (*Gcg*^gut−/−^) or only the distal gut (*Gcg*^distalgut−/−^) (*41*) (Fig. 6E). Total GLP-1 levels were enhanced both in 30- and 200-nm RM LNC–treated WT *Gcg*^+/+^ mice, yet the stimulatory effect was lost in *Gcg*^gut−/−^ mice (Fig. 6F), confirming the intestinal origin of the GLP-1 response. In *Gcg*^distalgut−/−^ mice, only 30-nm RM LNC triggered an elevation of GLP-1, whereas 200-nm RM LNC were ineffective (Fig. 6F). This suggests that small-diameter RM LNC interact with enteroendocrine L cells located in the upper intestine, whereas large-diameter RM LNC mainly interact with enteroendocrine L cells located more distally. This is consistent with the findings that gut hormone responses were more pronounced at early time points after gavage and that the small-diameter RM LNC were effective stimuli of GIP release, which arises from enteroendocrine K cells located predominantly in the proximal intestine (Fig. 6A). One plausible explanation to the differences encountered on gut hormone secretion between both nanoparticles exhibiting different sizes/compositions would be differences in digestion behavior. Glyceride lipids are digested primarily by pancreatic lipases in the upper small intestine to liberate fatty acids. We conducted in vitro digestion studies (fig. S26), measuring fatty acid liberation by both titration and gas chromatography, confirming a lag time in digestion with the 30-nm nanocapsules compared to the 200-nm capsules which were digested immediately after introduction of the lipase (*42*). The slower digestion of 30-nm LNCs could be due to the surfactant concentration in the 30-nm formulation, which was significantly higher than that of the 200-nm formulation. The presence of surfactants in dispersed lipid systems can cause a lag time for digestion (*43*). The different digestion profiles between different nanoparticles could explain the differences on hormone secretion ability between both nanoparticles.

Previous studies have demonstrated that modifying the particle size of nanocarriers may change their in vivo properties (*22*, *44*). Less, however, is known about whether, e.g., concentrating the nanocarriers and/or replacing excipients in the preparation procedure can alter the behavior of the nanocarriers upon in vivo administration. To evaluate the influence of excipient quality and quantity on the ability of lipid nanocarriers to stimulate gut hormone secretion, we analyzed total GIP levels, active GIP levels, active GLP-1 levels, and total PYY levels in plasma samples of mice receiving either concentrated lipid-based nanocarriers (Fig. 6, G to J) or nanocarriers where lipid excipients were replaced by other functional lipidic excipients with different lipid chain length (e.g., replacing C8 by C18) (Fig. 6, K to N; fig. S27; and table S9). The multibiological effects induced by concentrated 200-nm RM LNC were similar to those induced by nonconcentrated 200-nm RM LNC, with inhibition of GIP release and increased secretion of GLP-1 and PYY (Fig. 6, G to J). By contrast, the release of gut hormones following administration of 30-nm RM LNC, concentrated by a modified preparation process (Fig. 6, G to J), differed from nonconcentrated 30-nm RM LNC (Fig. 6, A to D), displaying inhibition of GIP, but no significant stimulatory effect on GLP-1 and PYY 1 hour after oral administration. In addition, 30- and 200-nm lipid nanocapsules with a lipid core of linseed oil (in place of Labrafac) and Peceol [LNC (Lin/Pec)] were also generated (table S9). Thirty-nanometer LNC (Lin/Pec) were similarly effective to 30-nm LNC (Lab/Pec) at stimulating GIP, GLP-1, and PYY release following oral administration (Fig. 6, K to N). After Labrafac lipophilic WL 1349 was replaced with linseed oil, 200-nm lipid nanocapsules had a similar effect on increasing GLP-1 and PYY (Fig. 6M and fig. S27) but, unlike those containing Labrafac, were stimulatory rather than inhibitory on GIP release (Fig. 6K).

### Therapeutic effect in HFD-induced diabetic/obese mice

Traditional methods to elevate plasma gut hormone activity, such as subcutaneous injections or oral administration of synthetic analogs, can be invasive or have limited efficacy. Tirzepatide, an injected GIP/GLP-1 coagonist, is the only US Food and Drug Administration–approved multitarget peptide combining activity against GIP and GLP-1 receptors. Oral semaglutide only mimics increased GLP-1 secretion and presents a low oral bioavailability (less than 1%) and large variability (*45*), a major shortcoming in oral peptide delivery. We evaluated the effect of lipid-based nanocarriers of different particle sizes (produced by different proportions of the same excipients in the formulations) loaded with the GLP-1 analog EXE, on gut hormone secretion and glucose tolerance in a HFD-induced obesity/diabetic model.

**Fig. 7.**
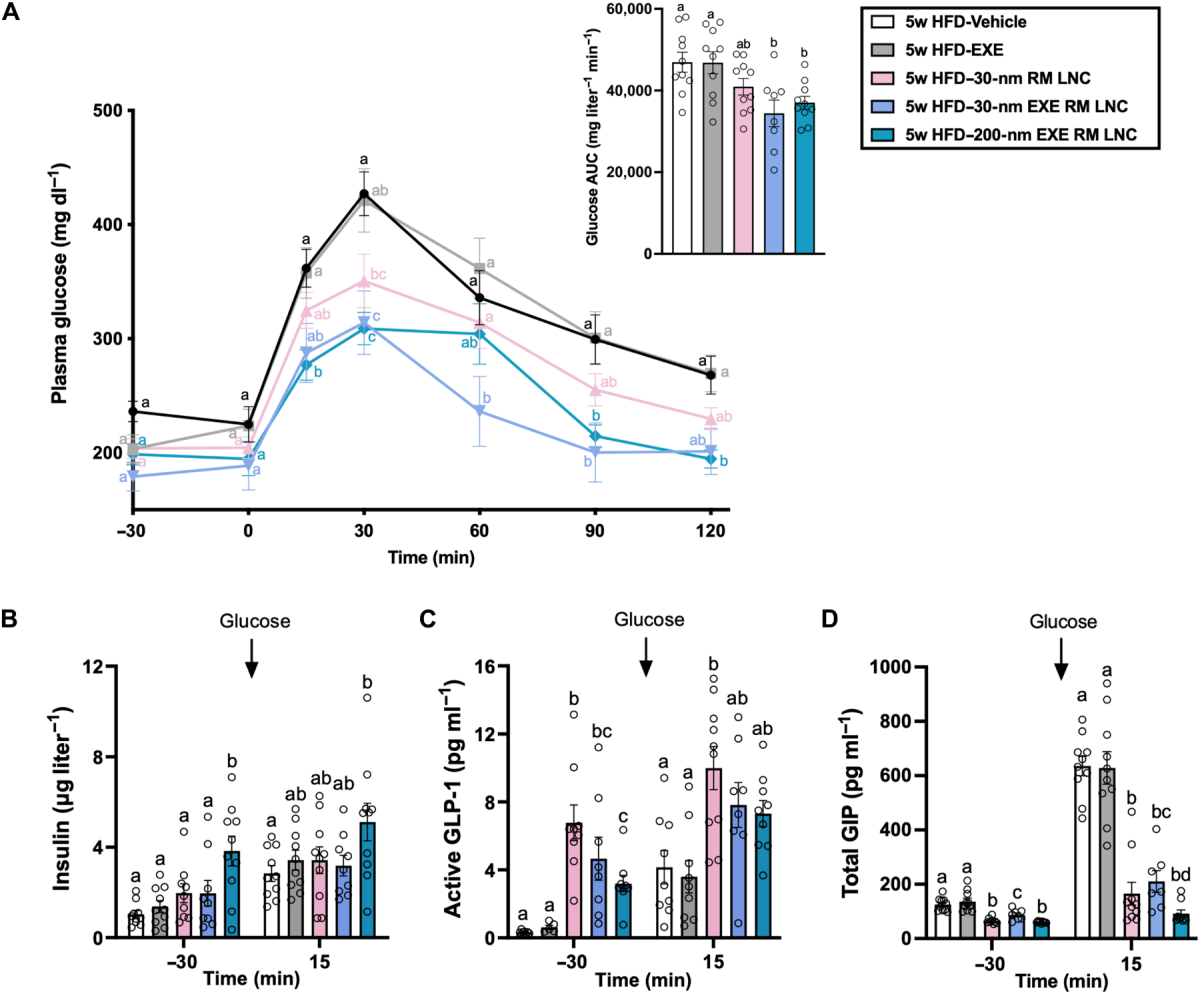
Lipid nanocarriers’ therapeutic effect in HFD-induced obese/diabetic mice. (**A**) Plasma glucose levels (mg dl^−1^) measured 30 min before and for 120 after oral glucose loading (2 g/kg) and mean glucose AUC (mg dl^−1^ min^−1^) throughout the glucose challenge (*n* = 8 to 10). (**B**) Plasma insulin levels (μg l^−1^) at 30 min before and 15 min after glucose challenge (*n* = 9 or 10). (**C** and **D**) Multibiological effects induced by lipid-based nanocarriers under pathological context or/and oral therapy of hormone analog. Plasma active GLP-1 levels (C) (*n* = 5 to 10) and plasma total GIP levels (D) (*n* = 7 to 10) quantified 30 min before and 15 min after glucose loading. Empty 30-nm lipid-based nanocarriers (1.62 mg/g lipid dose), EXE-loaded formulation (30-nm EXE RM LNC and 200-nm EXE RM LNC, 500 μg/kg EXE dose, 1.62 mg/g lipid dose), EXE in solution (500 μg/kg), or equivalent volume of water (vehicle) were orally administered 60 min before the oral glucose challenge. Data shown are mean, and data with different superscript letters are significantly different (*P* < 0.05) according to one-way or two-way ANOVA followed by Tukey’s post hoc test.

Mice received a single oral administration of an empty nanocarrier (30-nm RM LNC), EXE-loaded formulations (30-nm EXE RM LNC and 200-nm EXE RM LNC), EXE in solution, or an equivalent volume of water 60 min before an OGTT (Fig. 7). The administered doses of lipids and GLP-1 analogs in mice were 1.62 g/kg and 500 μg/kg, respectively. Oral glucose gavage (2 g/kg) was given at time = 0 min. The plasma glucose profile and glucose AUC of EXE solution–treated mice were not significantly different from vehicle controls. Conversely, EXE-loaded formulations (including 30-nm EXE RM LNC and 200-nm EXE RM LNC) lowered the blood glucose levels and glucose AUC compared with the vehicle-treated group (Fig. 7A). Insulin release was significantly stimulated following 200-nm EXE LNC administration, both before and after the glucose gavage (Fig. 7B). Compared with untreated mice, mice receiving empty 30-nm RM LNC had lower blood glucose levels 30 min after the oral glucose challenge (Fig. 7A), consistent with the previously observed regulation of blood glucose by blank 200-nm RM LNC (*18*).

Thirty minutes after oral administration of the nanocarriers (−30), HFD mice receiving empty lipid-based nanocarriers exhibited more than 20-fold higher levels of active GLP-1 than mice receiving water (Fig. 7C). Inclusion of EXE in the lipid nanocarriers resulted in a small stimulation of GLP-1 release (increased 14-fold in 30-nm EXE RM LNC and 9-fold in 200-nm EXE RM LNC versus untreated HFD) (Fig. 7C). A fivefold increase in GIP levels was observed 15 min after the glucose challenge in untreated and EXE solution–treated HFD mice (from ~130 to ~630 pg ml^−1^) (Fig. 7D). Conversely, all lipid-based nanocarriers, including the empty ones, induced a strong suppression of GIP levels before and after the glucose challenge in the HFD mice (Fig. 7D). The stimulatory ability of lipid-based nanoparticles on PYY release turned to inhibition with the addition of EXE in obese/diabetic mice (fig. S28), matching the lower release of GLP-1 in this group compared with unloaded RM LNC and consistent with studies indicating the exogenous GLP-1 decreases L cell secretion in individuals (*46*, *47*).

In this study, we investigated the therapeutic effect of lipid-based nanocarriers in T2DM mouse models presenting obesity. These results showed that the elevated endogenous gut hormones induced by the nanocarriers, e.g., GLP-1, exerted a better blood glucose control in mice. It has been reported that it is difficult for GLP-1RA drugs to exert an effect on weight loss in obese patients with T2DM (*48*–*50*). We also found no effect on weight loss after a chronic and long-term treatment in HFD-induced diabetic/obese mice in our previous study. Oral delivery of short-acting GLP-1RA (EXE) combined with increased levels of endogenous gut hormones did not meet the need for weight loss therapy (*18*). To this end, we have recently achieved a better weight loss when replacing EXE with a long-acting GLP-1RA (semaglutide) within the lipid nanocarriers (*51*). Hence, longer half-life peptides and/or prolonged treatments which would raise concentrations to levels sufficient to engage central appetite-regulating neurons might be needed toward an effect on body weight loss.

## DISCUSSION

Bariatric surgery is now the most effective intervention for inducing weight loss among individuals with obesity, which likely reflects at least in part the associated elevated postprandial concentrations of GLP-1 and PYY (*52*). Strategies to harness the translational potential by cotargeting more than one gut hormone receptor offer great hope for the treatment of T2DM and obesity. The value of such approaches has been demonstrated by positive outcomes in clinical trials of GLP1-based dual and triple agonist therapies (*12*, *53*, *54*). Combination of a GLP-1 agonist and a GIP antagonist seems also to have a therapeutic effect in preclinical models (*13*, *14*).

EECs regulate the secretion of gut hormones by interacting with gastrointestinal (GI) luminal nutrients (lipids, carbohydrates, proteins, etc.) (*55*). Many studies have highlighted the key role of regulating EEC secretion ability in improving metabolic dysfunction (*15*, *56*). Although nutritional strategies, such as dietary interventions and nutrient manipulations, have been widely studied to increase the endogenous release of gastrointestinal hormones in the field of nutrition and metabolism, the utilization of these strategies alone cannot meet the clinical needs of most patients with obesity and diabetes (*16*). Moreover, metabolic disorders have been a strong focus of efforts to deliver oral peptide therapies (*57*). However, current oral drug delivery systems often only focus on the delivery vehicle as a mean to protect drugs toward a better systemic absorption. Lipid excipients are widely used in the construction of drug carriers. Our previous studies have indicated that lipid-based nanoparticles can trigger the secretion of gut hormone GLP-1 and that this effect can also be synergistic in the treatment of incretin-based diabetes via oral peptide therapy (*17*–*19*). All excipients used in the formulation are GRAS, and no significant physiological toxicity was found after a 1-month daily oral administration in different mouse disease models (*18*, *58*). Notably, only lipid nanocapsules and ingestible electroceutical capsules have been shown to regulate the endogenous release of GI hormones, and both can only increase a single GI hormone (GLP-1 or ghrelin) (*17*, *59*). In this study, we exploit lipid nanocarriers to achieve smart noninvasive oral regulation of gut hormones (including the elevation and/or suppression of different intestinal hormones) per se, potentially mimicking the therapeutics of gut hormone coagonists or antagonists. We have found that different physiological effects can be achieved with the same type of nanocarrier, based on a different nanocapsule composition, the type of excipient, the preparation method, and the physicochemical properties of the nanocarriers. Namely, we found that a lipid-based nanocarrier can not only achieve increased endogenous release of various gut peptides (GLP-1, GIP, and PYY) secreted from different EECs (K cells and L cells) but also can be modified to simultaneously elevate endogenous GLP-1 and PYY release while suppressing GIP release, which has not been reported even in widely studied nutritional strategies on regulating release of GI hormones. This lipid-based nanocarriers can additionally deliver encapsulated drugs in a patient-friendly, inexpensive, and noninvasive manner (e.g., oral route).

This work introduces mouse and human intestinal organoids to study the regulation of gut hormones in the field of nanomedicine and drug delivery systems. With the advantages of knock-in and KO intestinal organoids, this study identifies molecular mechanisms that modulate gut hormone release induced by lipid-based nanocarriers, suggesting a potential strategy to combine nanotechnology and nutrients (such as lipids) with the purpose of selectively manipulating GPCRs expressed on EECs. In the past 20 years, previously orphan GPCRs expressed on EECs, such as GPR40, GPR120, GPR41, and GPR43, have been reported to be activated by fatty acids with various carbon chain lengths, thereby regulating the release of gut hormones (*21*). Many excellent studies have shown the structures of these receptors and how nutrients (such as lipids) and GPCRs regulate the release of intestinal hormones in the body, which has helped researchers develop strategies that can selectively target EEC GPCRs in the treatment of metabolic diseases (*60*, *61*). In this study, we found that the lipid-based nanocarriers per se can act on GPR40 and GPR119 (which is activated by monoacylglycerides), opening a notable chapter in the combination of nanotechnology and nutrients. In previous rodent studies evaluating the use of prebiotics toward GLP-1 secretion, prebiotics have been typically administered at a 10% dose in the diet (200 to 300 mg/day) (*62*); however, in the case of human clinical trials, the dose was 16 g/day (*63*, *64*). Hence, the allometric scaling of the dose of prebiotics toward GLP-1 secretion from mice to humans appears to be nonlinear. The doses of lipid nanocapsules needed toward gut hormone stimulation in vivo in humans will need to be evaluated toward their translation into the clinics.

We have demonstrated differences in gut hormone secretion triggered by nanoparticles in vivo in normoglycemic mice and identified differences in the origin of hormone secretion (duodenum versus distal gut) using KO mice. Differentially modulating the secretion of hormones from distinct regions of the gastrointestinal tract could be achieved by modifying the composition of the formulation. We believe that this is remarkable and paves the way for future research into modulating gut hormone secretion by drug delivery systems.

## METHODS

### Lipid-based nanocarrier preparation and characterization

Lipid-based nanocarriers, RM LNCs, were formulated as previously reported (*18*). Briefly, formulations were produced in two steps, in which formulated reverse micelles were further incorporated into lipid nanocapsules. Reverse micelles were formulated by mixing of 100 mg Span 80 (surfactant), 500 mg Labrafac WL 1349 (oil), and 50 μl of Milli-Q water (or peptide solution in water). Lipid nanocapsules were prepared by using a modified phase inversion (PIZ) process as described before (*18*). All excipients in the formulations (shown in table S1) were mixed under magnetic stirring. Three temperature cycles of progressive heating/cooling were conducted (from 40° to 65°C). In the last cycle, 500 μl of preheated reverse micelles was added into the mixture at around 2°C above the PIZ zone as shown in table S1. After that, 2500 μl of cold water was added during the cooling step at the PIZ zone under a high-speed stirring. The formulations were filtered using a 0.45-μm filter and stored at 4°C until use. Lipid nanocarriers presenting different particle sizes, including 30, 50, 150, and 200 nm, were obtained by changing the ratio of formulated excipients (shown in table S1). Concentrated formulations, including 30- and 200-nm RM LNCs, were prepared by adding 500 μl of cold water (instead of 2500 μl) in the last step during the preparation. Lipid-based nanocarriers with a lipid core of linseed oil were also obtained by replacing Labrafac WL 1349 with linseed oil.

Particle size and polydispersity index were measured by dynamic light scattering using a Zetasizer Nano ZS (Malvern Instruments Ltd., Worcestershire, UK). Zeta potential was also determined using a Zetasizer Nano ZS by laser Doppler velocimetry. Each measurement was conducted in triplicate. For EXE-loaded lipid nanocarriers, they were also characterized on the basis of the peptide encapsulation efficiency (EE) as previously described (*18*, *19*). Briefly, total peptide concentration was measured in lipid nanocarrier–dissolved methanol (dilution factor, 1:19). The free peptide was separated by an ultrafiltration method (dilution factor, 1:2). The EXE incorporated into the lipid-based nanocarriers was quantified by high-performance liquid chromatography (*18*, *19*). A gradient system was used, with an initial ratio of 10:90 (v/v, aqueous:organic phase) and a flow rate of 1 ml /min, with a linear change of 90:10 (v/v) in 10 min and a constant change in the next minute. Then, over the next 1.5 min, the ratio changed linearly to the initial composition and remained stable at the last minute. The aqueous mobile phase consisted of 0.05% (v/v) trifluoroacetic acid in water, and the organic mobile phase consisted of 0.05% (v/v) in acetonitrile. A Kinetex EVO C18 column (100 Å, 2.6 μm, 150 mm by 4.6 mm) (Phenomenex, USA) and a security guard column (Phenomenex, USA) were used at room temperature. Twenty microliters of samples was injected and detected under a wavelength of 220 nm. The retention time was 5.9 min, and the limits of detection and quantification were 1.1 ± 0.4 μg/ml and 3.3 ± 1.1 μg/ml, respectively. The EE was calculated using the following equation: EE (%) = (total amount of EXE − free EXE)/(total amount of EXE) × 100.

### Nanoparticle digestion studies

Digestion of lipid nanocarriers was measured using an in vitro lipolysis model. Nanoparticle dispersions (30 and 200 nm, 20 ml) were added to a thermostated glass vessel (37°C) at an equal total lipid content (40 mg/ml). The pH was adjusted to 6.500 using minimal volumes of 1 M NaOH or HCl solutions. Pancreatin suspension [~1000 tributyrin (TB)U/ml of digest] was prepared by suspension of the pancreatin powder [from porcine pancreas 4× US Pharmacopeia (USP) specifications] in tris-maleate buffer (50 mM, pH 6.5) with calcium chloride dihydrate (5 mM) and sodium chloride (150 mM), vortex mixing for 10 min to disperse the pancreatin powder, and centrifuging at 4°C for 15 min at 3500 rpm to collect the supernatant. Upon addition of the pancreatin supernatant (2 ml) to the nanoparticle sample, the pH of the digestion medium was maintained at pH 6.5 by titration of 1.0 M NaOH solution under the control of a pH stat control module (Metrohm AG, Herisau, Switzerland). Samples (200 μl) were retrieved at 0, 5, 10, 15, 20, 25, and 30 min for Gas Chromatography with Flame Ionization Detection (GC-FID) analysis of fatty acid composition. The lipolysis process was halted by adding 50 mM 4-Butylphenylboronic acid (4-BPBA) in methanol, stopping enzyme activity. Lipids were extracted with dichloromethane, redissolved in pentane, and converted to fatty acid methyl esters using methanolic HCl. The liberated fatty acid was then quantified by injection of 1 μl onto an Agilent 7820A GC with a FID detector module from Agilent Technologies (Glostrup, Denmark) on an Omegawax 320 column with a nitrogen flow at 0.4 ml/min and an injector temperature set to 250°C. A ramped oven temperature program was used with a starting temperature of 40°C (held for 1 min) increased by 3°C/min to 200°C (held for 10 min) and then increased by 3°C/min to 260°C. The FID was set to 250°C with an airflow of 350 ml/min, a hydrogen flow of 35 ml/min, and a makeup gas flow of 40 ml/min. All reagents for digestion experiments were purchased from Sigma-Aldrich (Soborg, Denmark), and water was from a PureLab flex system from ELGA LabWater (High Wycombe, UK), with a resistance of 18.2 megohm.

### Murine enteroendocrine L cell line culture

GLUTag cells (murine enteroendocrine L cells) were donated by D. J. Drucker (Lunenfeld-Tanenbaum Research Institute, University of Toronto, Canada), and passages 10 to 15 were used in these studies. Murine L cells were grown in 0.05% (v/v) Matrigel-coated flasks and low-glucose Dulbecco’s modified Eagle’s medium (DMEM; Sigma-Aldrich) supplemented with 10% (v/v) inactivated fetal bovine serum (FBS; Gibco) and penicillin/streptomycin (100 U ml^−1^; P/S) (Sigma-Aldrich), at 37°C with 5% CO_2_ supply. Cells were passaged every 4 to 6 days using trypsin (0.25%) containing EDTA (0.02%) digestion. Cells (0.5 × 10^6^ cells ml^−1^) for experiments were plated on 2% Matrigel-coated 24-well plates for secretory studies (*65*).

### Organoid culture media

Two base mouse organoid media, called ENR[containing epidermal growth factor, noggin, and R-spondin 1 (RSPO1)] medium and WENR medium (Wnt3A-conditioned medium to ENR culture medium), were used (*66*, *67*). ENR medium is advanced DMEM/F12 (ADF, Invitrogen) supplemented with 10% RSPO1-conditioned medium, P/S (100 U ml^−1^), murine noggin (100 ng/ml; PeproTech), murine epithelial growth factor (EGF; 50 ng/ml; Invitrogen), 2 mM l-glutamine (Sigma-Aldrich), 1× N2 (Invitrogen), 1× B27 (Invitrogen), 1 mM *N*-acetyl-l-cysteine (NAC; Sigma-Aldrich), and 10 μM Rho-associated protein kinase (ROCK) inhibitor Y-27632 (Tocris). WENR medium is an ENR medium containing an additional 50% Wnt3A-conditioned medium. Mouse duodenal, jejunal, and ileal organoids were cultured and differentiated in ENR medium. Intestinal crypt culture was performed in WENR medium before and after electroporation.

IFE and IF media were used in human intestinal organoid culture as previously reported (*68*). Both media were prepared in ADF medium with supplements of 40% Wnt3A-conditioned medium, 10% RSPO1-conditioned medium, P/S (100 units ml^−1^), murine noggin (100 ng/ml), 2 mM l-glutamine, 1× N2, 1× B27, 1 mM NAC, 10 μM ROCK inhibitor Y-27632, 500 nM A83-01 (Tocris), and 10 nM human [Leu^15^]–gastrin I (Sigma-Aldrich). Unlike IF medium, IFE medium contains an additional murine EGF (50 ng/ml). IFE medium was used for human intestinal crypt culture, and IF medium was used for EEC differentiation.

RSPO1-conditioned medium was produced using human embryonic kidney 293 cells transfected with R-spondin-1 (Trevigen) (*68*). Cells were cultured in DMEM (Gibco) supplemented with 10% (v/v) FBS, P/S (100 units ml^−1^), 1% GlutaMAX (Gibco), and zeocin selection antibiotic (0.3 mg ml^−1^; Gibco), at 37°C with 5% CO_2_ supply. Zeocin was removed from culture for 3 days, and then cells were reseeded in ADF supplemented with 10% (v/v) FBS, P/S (100 units un^−1^), 1 mM Hepes, and 1% GlutaMAX. Conditioned medium was harvested and purified after culture.

Wnt3A-conditioned medium was produced using L-M(TK-) cells transfected with a Wnt3A expression [American Type Culture Collection (ATCC) CRL-2647] (*68*). Cells were grown in DMEM supplemented with 10% (v/v) FBS, P/S (100 units ml^−1^), and G418 selection antibiotic (0.4 mg/ml; Sigma-Aldrich), at 37°C with 5% CO_2_ supply. G418 was removed from culture for Wnt3A-conditioned medium. Conditioned medium was harvested and purified after culture.

### Mouse organoid culture

Duodenal, jejunal, and ileal mouse organoid line generation, culture, and maintenance were performed as previously described (*68*, *69*). Briefly, duodenal organoids were generated from isolated duodenal tissues (proximally 3 cm beyond the stomach) from transgenic GIP-Cre/ROSA26-tdRFP mice (Fig. 1A) expressing a fluorescent marker in GIP-positive cells (enteroendocrine K cells) (*70*). Jejunal organoids were established from jejunal tissues (proximally 10 to 20 cm beyond the stomach) from male C57BL/6J mice. Ileal organoids with observable enteroendocrine L cells were generated from isolated ileal tissues (distal 10 cm to the ileal-caecal junction) from mice transgenically expressing a fluorescent reporter (Epac2-camps) under the control of the proglucagon (Gcg) promoter (Fig. 1A) (*71*). Isolated and washed tissues were chopped into 3- to 5-mm fragments and incubated in 30 mM EDTA in phosphate-buffered saline (PBS) for 5 min, with tissue shaken in PBS after each EDTA treatment to release intestinal crypts (*68*, *69*). The collected crypts were further purified by filtering through a 70-μm cell strainer coated with FBS (Thermo Fisher Scientific). The remaining crypts were resuspended into basement membrane extract (BME; R&D Technology) and were seeded into 12-well plates (Corning), with 15-μl domes polymerized for around 45 min at 37°C (*68*, *69*). Mouse intestinal organoid medium ENR with 10 μM ROCK inhibitor Y-27632 (Tocris) was then overlaid and replaced every 2 to 3 days. Mouse organoids were passaged every 10 to 12 days using TrypLE digestion for 3 min at 37°C, followed by mechanical shearing to break organoids into small clusters and then resuspended in BME as before (*68*, *69*).

### Human organoid culture

Human duodenal and ileal organoids were generated from anonymized surgical specimens [Tissue Bank, Addenbrooke’s Hospital (Cambridge, UK)], and ethical approval was approved by East of England–Cambridge Central Research Ethics Committee with license number 09/H0308/24. Human duodenal and ileal organoid line generation, culture, maintenance, and differentiation were conducted as previously reported (*66*, *68*). Briefly, tissue pieces were incubated with 30 mM EDTA for 3 × 10 min, with tissue shaken in PBS after each EDTA treatment to obtain isolated crypts. After purifying isolated intestinal crypts, they were further digested in TrypLE for 3 min at 37°C to generate small cell clusters. Cell clusters diluted in BME were then seeded in 12-well plates as small domes (approximately 15 μl per dome), which was polymerized at 37°C for around 45 min. Culture medium (IFE) was then overlaid to cover the domes and changed two times per week. Human intestinal organoids cultured in IFE medium were passaged every 14 to 21 days using TrypLE incubation for 15 min at 37°C to open clumps and were then mechanically sheared with rigorous pipetting to get small clusters. These were resuspended in BME and seeded into multiwell plates as before. To successfully obtain differentiated EECs, EGF was removed from culture once organoids formed large budded structures in IFE medium, with subsequent culture in IF medium for a further 10 to 14 days. For accelerating enteroendocrine differentiation, Wnt3A-conditioned media was reduced to 10% in IF medium, with supplements of 10 μM N-[N-(3, 5-difluorophenacetyl)-l-alanyl]-s-phenylglycinet-butyl ester (DAPT, Notch inhibitor, Generon) and 100 nM PD0325901 (MEK inhibitor, Sigma-Aldrich). To enable identification of human K cells and L cells, transgenic human organoid lines (duodenal GIP-Venus organoid line and ileal GCG-Venus organoid line) were generated as previously reported (*72*).

### Mouse nutrient sensor KO intestinal organoid line generation

Different GPCR KO organoid lines were generated by CRISPR-Cas9 gene editing. Cas9-GFP mouse ileal organoids were derived from the isolated intestines of Rosa26-Cas9 knock-in mice (*73*) with Cre recombinase–dependent Cas9 endonuclease expression, 3xFLAG epitope tag, and CMV early enhancer/chicken β actin (CAG) promoter–directed enhanced green fluorescent protein (EGFP; Fig. 2A). The expression of Cas9 and EGFP is blocked by the upstream Lox-stop-Lox sequence. After the generation of organoids, the activation of Cas9 expression was obtained by deletion of the loxP-flanked stop element upon treatment with the TAT-Cre protein.

A pair of sgRNAs targeting transmembrane helices 1 and 2 in exon 1 of *Ffar1* gene (TTGAACTTGTTAGCCATCCG and TGGAGAGTGTAGACCAAGCT; fig. S11 and table S5) were designed (www.idtdna.com) and synthesized (Merck, UK). Each sgRNA (5 μM) was delivered to dissociated Cas9-GFP mouse ileal organoids by electroporation. Differentiated organoids were manually picked to establish clonal organoid lines 14 to 21 days post-electroporation. DNA of picked clonal lines was extracted using QuickExtract DNA Extraction Solution (Lucigen Corporation, USA), with successful deletion measured by PCR screening using a pair of primers (table S6) and a secretion assay (FFAR1 agonist AM1638, Generon, USA).

GPR119 KO organoids were obtained by using a pair of sgRNAs targeting transmembrane helices 1 and 3 in exon 1 of *Gpr119* (AAGGATCACTCCAAATGAGA and GACCTTGTGTAGCCTTCGGA; fig. S12 and table S5). The PCR-screened KO clonal line was also confirmed by a secretion assay using the GPR119 agonist AR231453 (Sigma-Aldrich, UK).

FFAR4 KO organoids were obtained by using a pair of sgRNAs targeting transmembrane helices 1 and 2 in exon 1 of *Ffar4* (TCGTGGAGACCACCGTTCTG and CACGACGAGCACTAGAGGGA; fig. S13 and table S5). The successful deletion was measured by PCR screening (table S6).

FFAR1 and GPR119 dual-KO organoids were generated by further deleting a functional region of *Ffar1* gene in previously obtained GPR119 KO organoids. The successful deletions in both genes were measured by PCR screening (table S6) and secretion assays (FFAR1 agonist AM1638 and GPR119 agonist AR231453).

### Human nutrient sensor KO intestinal organoid line generation

Human ileal FFAR1 KO and GPR119 KO organoids were generated by CRISPR-Cas9–mediated nonhomologous end joining. Guides targeting human *FFAR1* transmembrane helices 2 and 4 (CTGGTCTACGCCCTGAACCT and CGGAAGGCTTGGTAGCCCAA; fig. S18 and table S7) and human *GPR119* transmembrane helices 2 and 3 (AGAGATGGCCACACCAATCA and AAGTGACAAATGCCATCCGC; fig. S18 and table S7), respectively, were designed and synthesized. The guides were cloned into px458-pSpCas9(BB)-2A-GFP CRISPR-Cas9 (a gift from F. Zhang, Addgene plasmid #48138) using BbsI (New England Biolabs) Golden Gate cloning (*74*). Guide plasmids were confirmed by Sanger sequencing (Source BioScience), amplified, and then purified using a HiSpeed Maxi Kit (QIAGEN). Purified plasmids were concentrated by ethanol precipitation and diluted in water to a final concentration above 2 μg μl^−1^. Twenty micrograms of each guide plasmid was transferred to dissociated organoids by electroporation (*75*). Differentiated organoids were manually picked to establish clonal organoid lines around 21 days post-electroporation. DNA was extracted using QuickExtract DNA Extraction Solution, with successful deletion measured by PCR screening using a pair of primers (table S8) and a secretion assay (FFAR1 agonist AM1638 or GPR119 agonist AR231453).

### 2D organoid monolayer culture

2D organoid monolayer culture was adapted from previously reported protocols (*68*, *72*). Briefly, well-established and well-differentiated mouse intestinal organoids were dissociated using the TrypLE reagent for 2 to 4 min at 37°C. Well-established and well-differentiated human intestinal organoids were dissociated by incubation in TrypLE for 10 to 15 min at 37°C. Dissociated organoids were seeded onto 2% Matrigel (Corning) precoated 48-well plates (secretion assays) or 96-well plates (cell toxicity assays). The plates were further incubated at 37°C (with a supplement of 5% CO_2_) for 18 to 24 hours before experiments.

### Cell toxicity assays

2D organoid monolayer cultured plates (96-well) were washed three times with 138 buffer (saline buffer) containing 138 mM NaCl, 4.5 mM KCl, 4.2 mM NaHCO_3_, 1.2 mM NaH_2_PO_4_, 2.6 mM CaCl_2_, 1.2 mM MgCl_2_, and 10 mM Hepes (pH 7.4). The cellular toxicity of lipid-based nanocarriers with different particle sizes, or different concentrations, was tested on 2D organoid monolayers using colorimetric assays for LDH and MTT. Tested formulations were dispersed in 138 buffer with a supplement of 1 mM glucose and 0.1% bovine serum albumin (basal buffer) and were incubated with cells at 37°C. After 2-hour incubation, 50 μl of the supernatant was collected and stored at 4°C for LDH analysis. The 2D organoid monolayers were washed twice with basal buffer followed by the addition of MTT solution (0.5 mg ml^−1^) in basal buffer to each well and incubated for 3 hours at 37°C. The MTT solution was then retrieved, and 200 μl of dimethyl sulfoxide was added to dissolve the formed purple formazin crystal. The absorbance of the plate was read at 560 nm using a microplate reader (Tecan, Infinite M1000 PRO). The previously collected 50-μl supernatant, mentioned above, was analyzed using a CyQUANT LDH Cytotoxicity Assay kit (Thermo Fisher Scientific), with measurements of the absorbance at 490 and 680 nm. Basal buffer media and 0.5% Triton X-100 in basal media were used as negative and positive controls, respectively. The negative controls were used to calculate the percentage of cellular viability in MTT assay. The empty formulations (no cells), negative controls, and positive controls were used to calculate LDH cytotoxicity.

### Secretion assays

Murine L cell, 2D mouse organoid monolayer, or 2D human organoid monolayer plates were washed three times with basal buffer for 30 min at 37°C. After pretreatment, cells were stimulated by addition of tested reagents, formulations, and excipients. After incubation at 37°C for 2 hours, supernatants were taken, remaining cellular debris were removed by centrifugation (2000*g*, 5 min, 4°C), and resulting supernatants were snap-frozen for further analysis. Lysate samples were obtained by lysing cells, scraping, and collecting cellular contents, followed by centrifugation of collected lysates at 10,000*g* for 10 min at 4°C. The resulting lysates were snap-frozen for further analysis. The release levels of tested hormones were calculated from the GLP-1 concentration measured in the supernatant divided by the total levels in the supernatant and the lysate and were normalized to a basal group to show as fold change.

For the experiment using the pharmacological inhibitor UBO-QIC, the plates were pretreated with UBO-QIC for 30 min at 37°C, before the addition of tested reagents, formulations, and excipients. Then, all tested groups were incubated with cells for another 2 hours at 37°C, in which the concentration of inhibitors was maintained constant.

### Live-cell imaging

Live-cell imaging of morphology of enteroendocrine L cells in the fluorescent gene knock-in organoid 2D monolayer was performed 2 hours after cells were treated with tested nanoparticles. Live cells in basal buffer or nanoparticle suspension were imaged on a CellDiscoverer 7 (Zeiss).

### Animals

All mouse experiments were approved by the UCLouvain Animal Committee under references 2018/UCL/MD/045 and 2021/UCL/MD/055 and the Animal Care and Use Subcommittee at the Toronto Centre for Phenogenomics, Mt. Sinai Hospital. Mice were housed up to five per cage, kept under a 12-hour light/12-hour dark cycle in the animal facility. All mice were given free access to food and water unless otherwise indicated. C57BL/6J male mice were obtained from Janvier Laboratories (France). *Gcg*^gut−/−^, *Gcg*^distalgut−/−^, and their littermate control (+/+) mice were generated and genotyped as previously reported (*41*). Studies were performed in animals aged 6.5 to 18 weeks and with the use of age- and sex-matched littermates.

### Hormone secretion in vivo

Mice were fasted overnight, and blood samples were collected at predesigned time points from the tail and portal veins after oral administration of tested formulations, excipients, and reagents. The blood collected per mouse in a given experiment was collected in heparin-coated capillary tubes at either 0 hours, 1 hour, and 3 hours or 0, 15, 30, 60, and 65 min after administration. For the measurement of intestinal hormones, including active GLP-1, total GLP-1, active GIP, total GIP, and total PYY, blood was mixed with the Dipeptidyl peptidase IV (DPPIV) inhibitor (20 μl per ml of blood), and plasma was isolated after centrifugation (3000 rpm, 10 min at 4°C). The isolated plasma was stored at −80°C until further analysis.

### Glucose tolerance test

#### Empty lipid-based nanocarriers and lipid mixtures

Male C57BL/6J mice were fed a HFD for 3 weeks and were fasted overnight before the study (the vehicle group was fed a CD and a HFD), empty 30-nm lipid nanocapsules (30-nm RM LNC) and lipid mixtures containing the same amounts of excipients present in 30-nm RM LNC (30 Mixture) (lipid dose: 1.62 mg/g).

#### Drug unloaded and loaded lipid-based formulations

Male C57BL/6J mice were fed a HFD for 5 weeks and were fasted overnight before oral gavage with water (vehicle group), free EXE peptide solution, empty 30-nm lipid-based nanocarriers (30-nm RM LNC), and 30- or 200-nm drug-loaded formulations (30-nm EXE RM LNC and 200-nm EX RM LNC) (500 μg/kg EXE dose, 1.62 mg/g lipid dose).

After 1-hour administration, an OGTT was carried out using 2 g/kg body weight of glucose. Blood glucose levels were assessed in tail vein blood using a hand-held glucometer (Accu-Chek, Roche, Switzerland), measuring the blood from the tip of the tail vein 30 min before the glucose load (−30 min) and 0, 15, 30, 90, and 120 min after glucose administration. Blood from the tail vein was collected at 30 min before the glucose load (−30 min) and 15 min after the glucose load (15 min) using heparin-coated capillaries and was mixed with the DPPIV inhibitor (20 μl per ml of blood). The plasma was isolated after centrifugation (3000 rpm, 10 min at 4°C) and stored at −80°C until further analysis.

### Hormone measurements

Total GLP-1 (Mesoscale), active GLP-1 (Mesoscale), mouse total GIP (Millipore), mouse active GIP (Crystal Chem), human total GIP (Mesoscale), mouse total PYY (Mesoscale), and human total PYY (Mesoscale) were measured in resulting supernatants, lysates, and plasma.

### DPPIV activity measurements

DPPIV activity was assessed as described previously (*76*). Briefly, the DPPIV activity was quantified by the production of para-nitroanilide (PNA) from glycine-proline-PNA (Sigma-Aldrich), within a standard curve of free PNA. Collected plasma samples were incubated for 30 min with Gly-Pro-PNA at 37°C, and an enzymatic activity was measured by kinetic analysis using a wavelength of 380 nm (SpectraMax M2; Molecular Devices, San Jose, CA, USA).

### Statistics

GraphPad Prism software was used for the statistical analysis. The Grubbs’ test for outlier detection in each group was conducted before all analyses. All data were represented as the mean ± SEM. For studies containing more than two groups, statistical analyses were conducted using one-way ANOVA followed by Tukey’s post hoc test or two-way ANOVA followed by a Sidak post hoc. Student’s *t* test or Mann-Whitney test was used in statistical analysis when only two groups. The threshold for statistical significance was *P* < 0.05.
