# Supplementary material for: Smart control lipid-based nanocarriers for fine-tuning gut hormone secretion
Source: Sci Adv. 2024 Dec 13;10(50):eadq9909. doi: 10.1126/sciadv.adq9909 (PMC11641013; doi:10.1126/sciadv.adq9909)
Supplement: Supplementary file 1 — Figs. S1 to S28 Tables S1 to S9 [file sciadv.adq9909_sm.pdf]

Science Advances
AAAS

Supplementary Materials for

Smart control lipid-based nanocarriers for fine-tuning gut hormone secretion

Yining Xu *et al.*

Corresponding author: Frank Reimann, fr222@cam.ac.uk; Fiona M. Gribble, fmg23@cam.ac.uk;
Ana Beloqui, ana.beloqui@uclouvain.be

Sci. Adv. **10**, eadq9909 (2024)
DOI: 10.1126/sciadv.adq9909

This PDF file includes:

Figs. S1 to S28
Tables S1 to S9

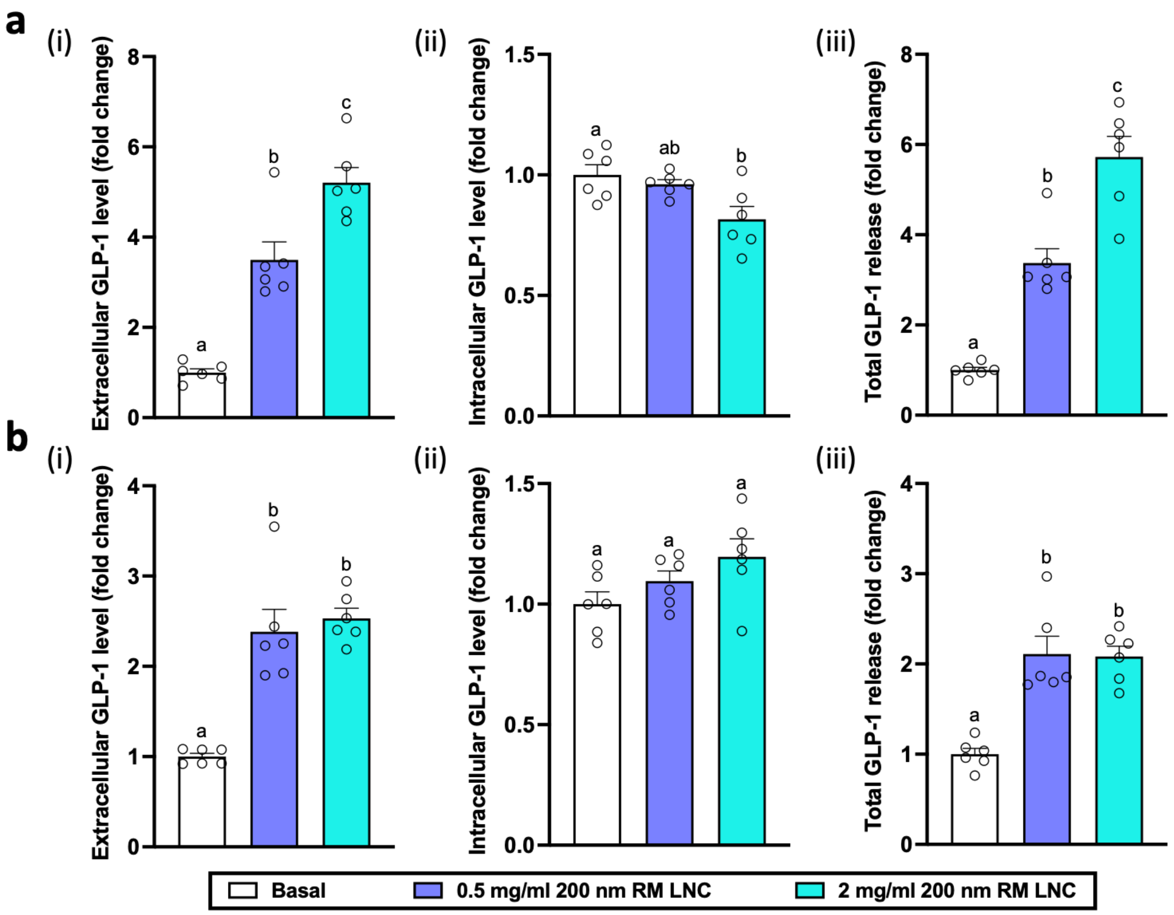

Fig. S1: GLP-1 secretion mediated by 200 nm RM LNC in ileal transgenic organoids. a,b, Total GLP-1 release (iii) from 2D monolayer of mouse GLU EPAC2-GFP ileal organoids (**a**) and human GLU GCG-Venus ileal organoids (**b**) was calculated from the total GLP-1 level in supernatant (i) divided by GLP-1 levels in the supernatant (i) and lysate (ii) measured by ELISA after 2 h co-incubation with nanoparticles (0.5 mg/ml and 2 mg/ml 200 nm RM LNC dispersed in 138 buffer containing 1 mM of glucose (basal buffer)). The release levels were normalized to basal group to show as fold change. For all figures, data are presented as mean ± SEM and n=6 replicates each from 3 independent experiments. Statistical comparisons (different superscript letters; $p<0.05$) were performed by one-way ANOVA followed by Tukey's post hoc test.

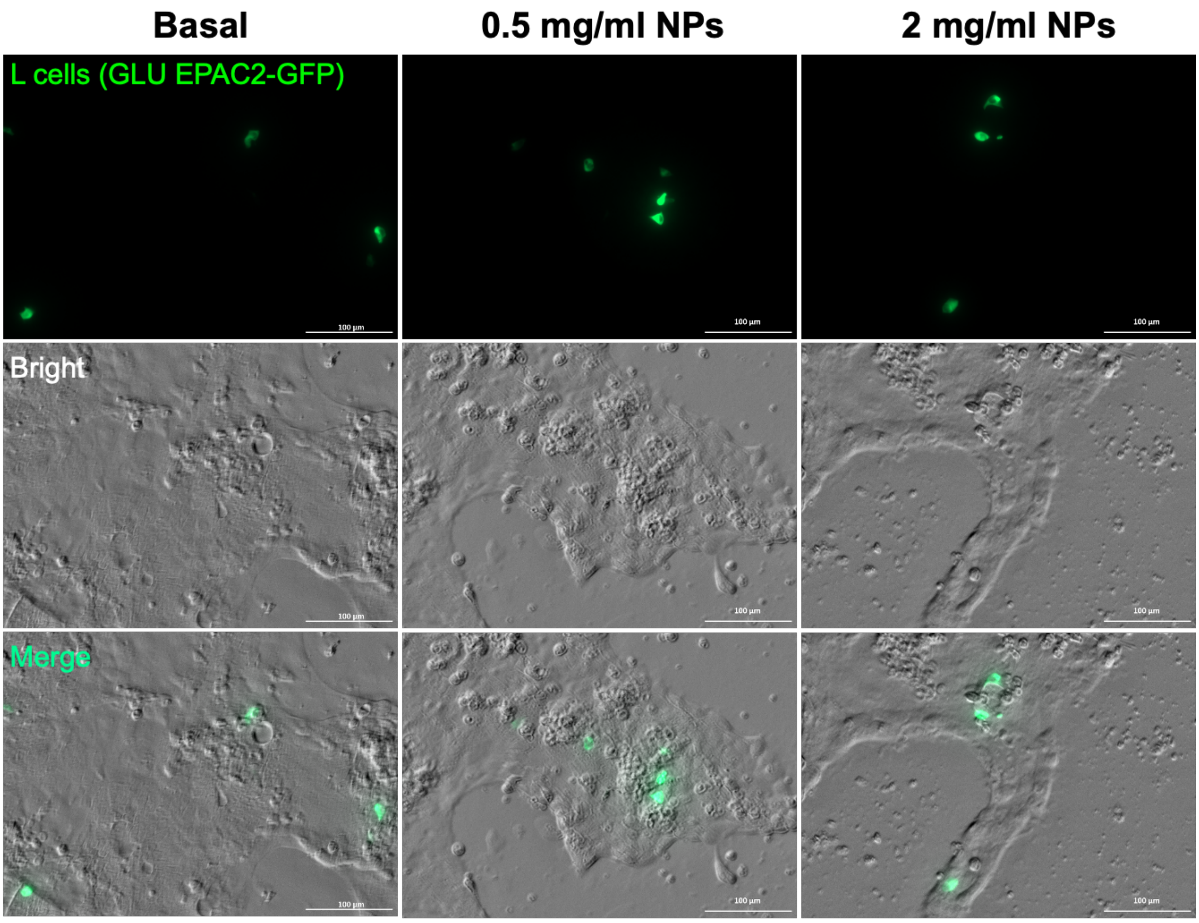

Fig. S2: Morphology of enteroendocrine L cells in mouse ileal transgenic organoids. Live images show the morphology of L cells (green) in mouse GLU EPAC2-GFP ileal organoids 2D monolayer after incubation with a suspension of nanoparticles (138 buffer containing 1 mM glucose (basal) supplemented with 0.5 mg/ml and 2 mg/ml 200 nm RM LNC). Representative images were selected from 3 independent experiments (n=6). Scare bar, 100 µm.

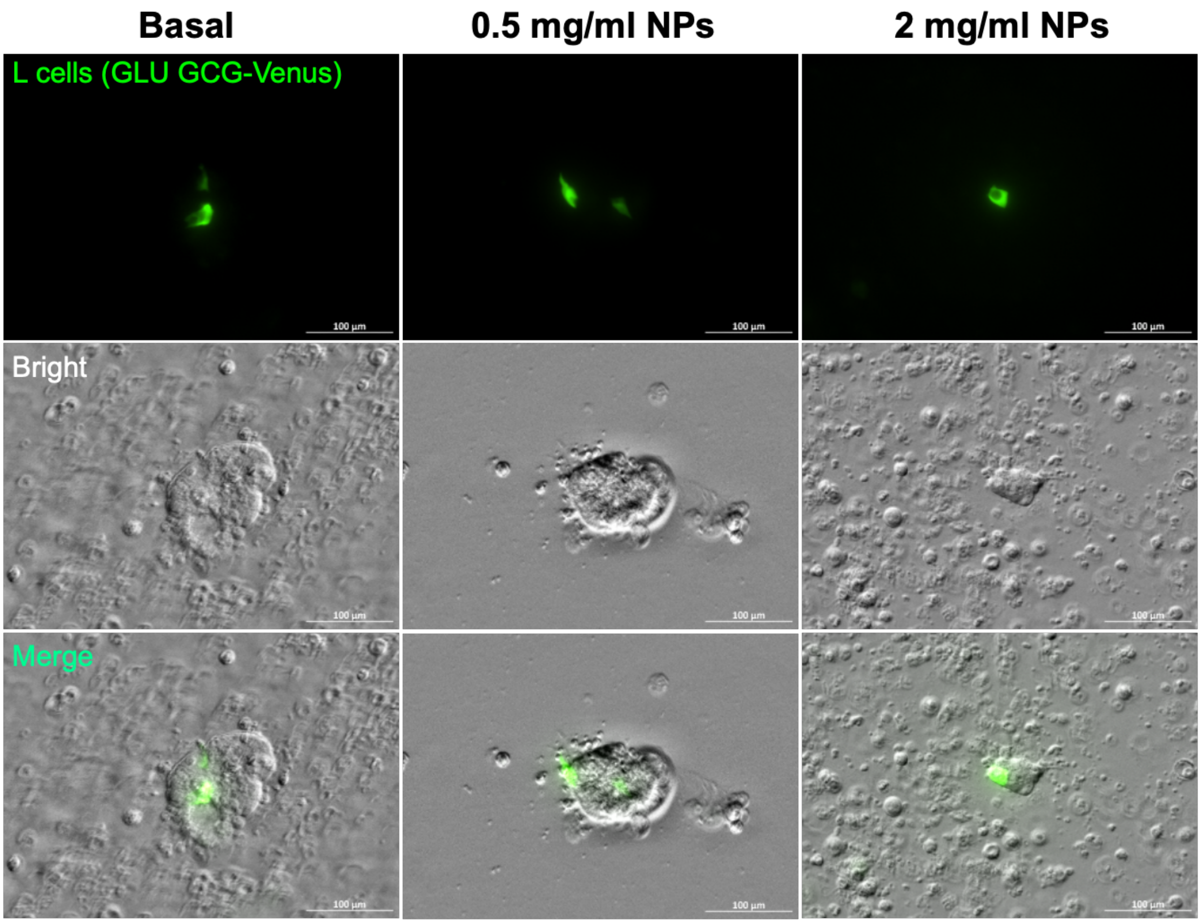

Fig. S3: Morphology of enteroendocrine L cells in human ileal transgenic organoids. Live images show the morphology of L cells (green) in human GLU-Venus ileal organoids 2D monolayer after incubation with suspensions of nanoparticles (138 buffer containing 1 mM glucose (basal) supplemented with 0.5 mg/ml and 2 mg/ml 200 nm RM LNC). Representative images were selected 3 independent experiments (n=6). Scare bar, 100 µm.

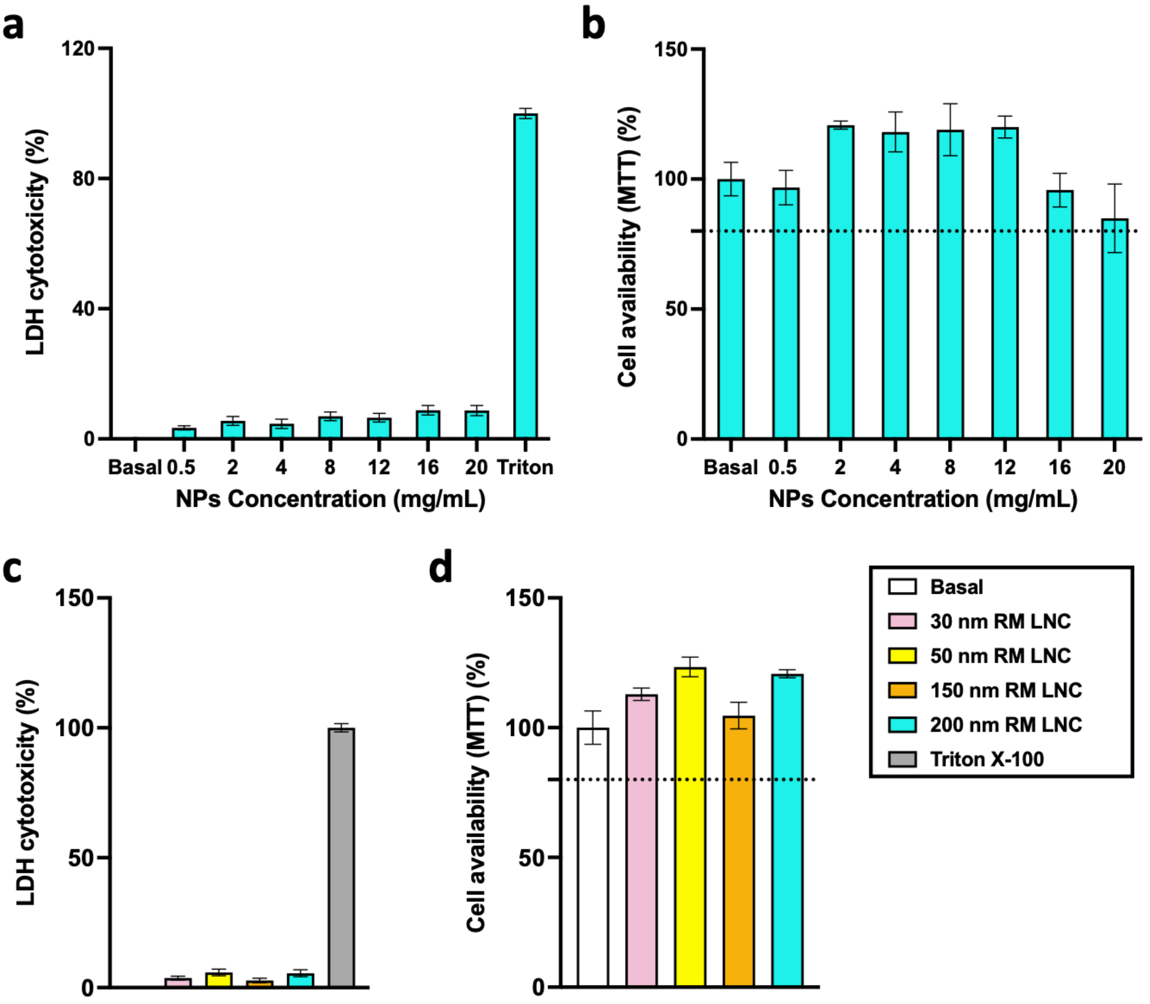

Fig. S4: Toxicity of reverse micelles-loaded lipid nanocapsules (RM LNC) in mouse intestinal organoids. a,b, Cell viability of mouse GLU-EPAC2-GFP ileal organoids with 200 nm empty RM LNC is detected by LDH (**a**) and MTT (**b**) assays, respectively, following treatment with 0.5 mg/mL ~ 20 mg/mL nanocapsules in basal buffer at 37 ° C for 2 h. **c,d,** Cell viability of mouse GLU-EPAC2-GFP ileal organoids was tested by conducting LDH (**c**) and MTT (**d**) assays after incubation with blank RM LNCS presenting different sizes (including 30 nm, 50 nm, 150 nm, and 200 nm) at 2 mg/ml nanoparticle suspension at 37 degrees for 2 h. In all data, basal (138 buffer containing 1 mM glucose) and 0.5% Triton X-100 in basal buffer were used as negative and positive controls, respectively. Negative controls were used to calculate the percentage cell viability. Data are shown as mean ± SEM (n=9 from 3 experiments).

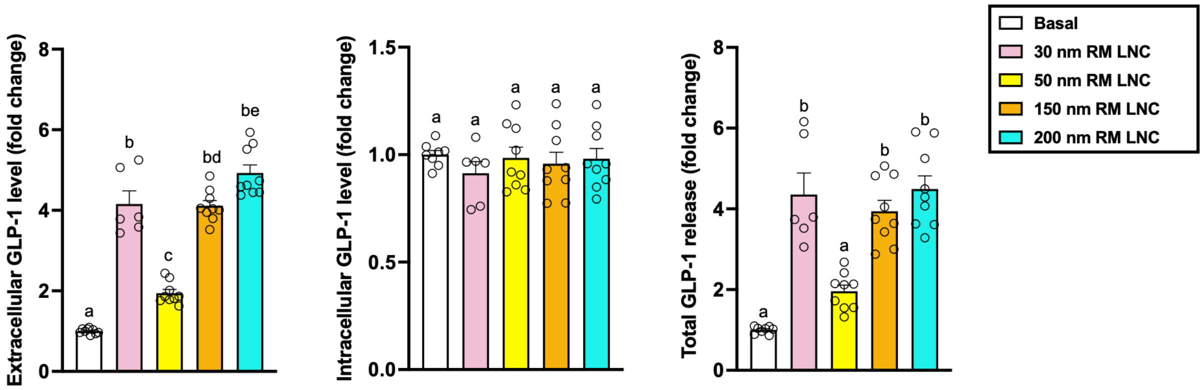

Fig. S5: RM LNC with different particle sizes stimulate GLP-1 secretion in murine L cells *in vitro*. GLP-1 release (right) calculated from the total GLP-1 levels measured in supernatants (left) and lysates (middle) by ELISA after RM LNC of different sizes (30 nm, 50 nm, 150 nm and 150 nm) were incubated with GLUTag cells at 37 ° C for 2 h. The release levels of total GLP-1 were calculated from the GLP-1 concentration measured in supernatant divided by total levels in the supernatant and the lysate, and were normalized to basal group to show as fold change. The nanoparticles are dispersed in 138 buffer containing 1mM glucose (base) as 2mg /ml. Data are expressed as mean ±SEM (n=6-9), corresponding to 3 independent experiments. Statistical comparisons (different superscript letters; $p<0.05$) were performed by one-way ANOVA followed by Tukey's post hoc test.

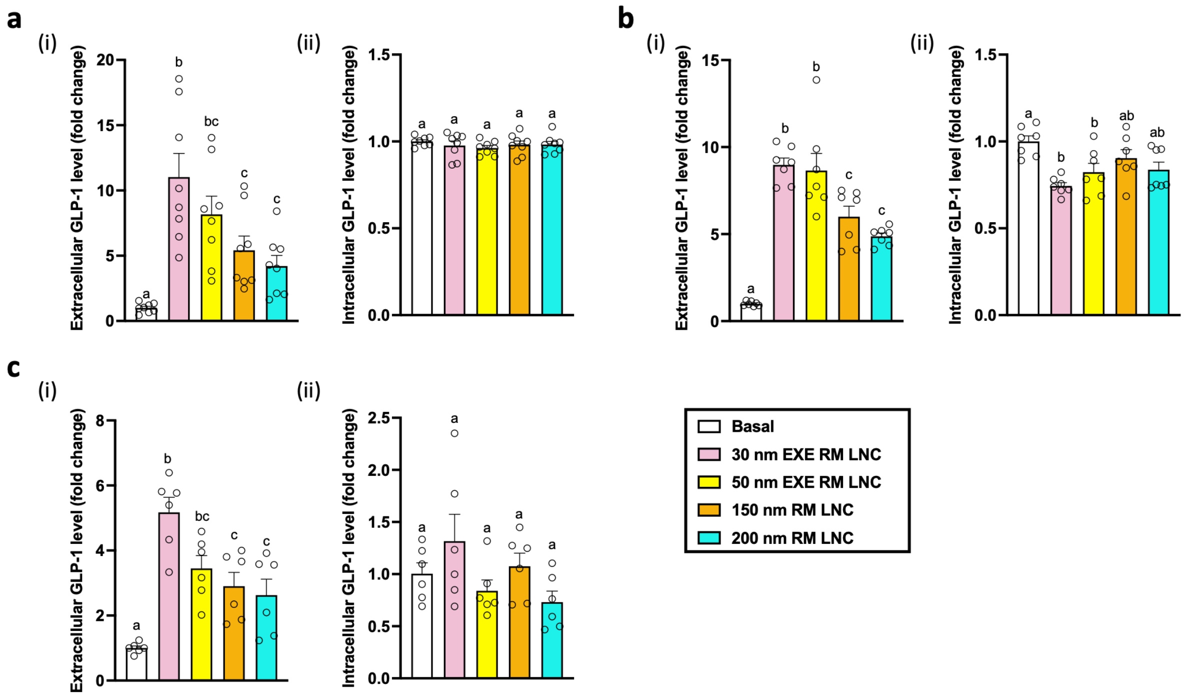

Fig. S6: RM LNC with different particle size stimulate GLP-1 secretion in mouse and human intestinal organoids *ex vivo*. a-c, Total GLP-1 level in supernatants (i) and lysates (ii) measured by ELISA after 2 h of coincubation with RM LNC of different sizes (from 30 nm to 200 nm) in 2D monolayer of mouse jejunal organoids (**a**), mouse GLU EPAC2-GFP ileal organoids (**b**) and human GLU-Venus ileal organoids (**c**). The tested nanoparticles are dispersed in 138 buffer containing 1 mM glucose (Basal buffer) at concentration of 2 mg/ml. All data are expressed as fold change of total GLP-1 level in organoids responding to basal buffer. Data with different superscript letters are significantly different ($p<0.05$), determined by a one-way ANOVA followed by Tukey's post hoc test. The data are presented as mean ± SEM (n=6-8), corresponding to 3-4 independent experiments.

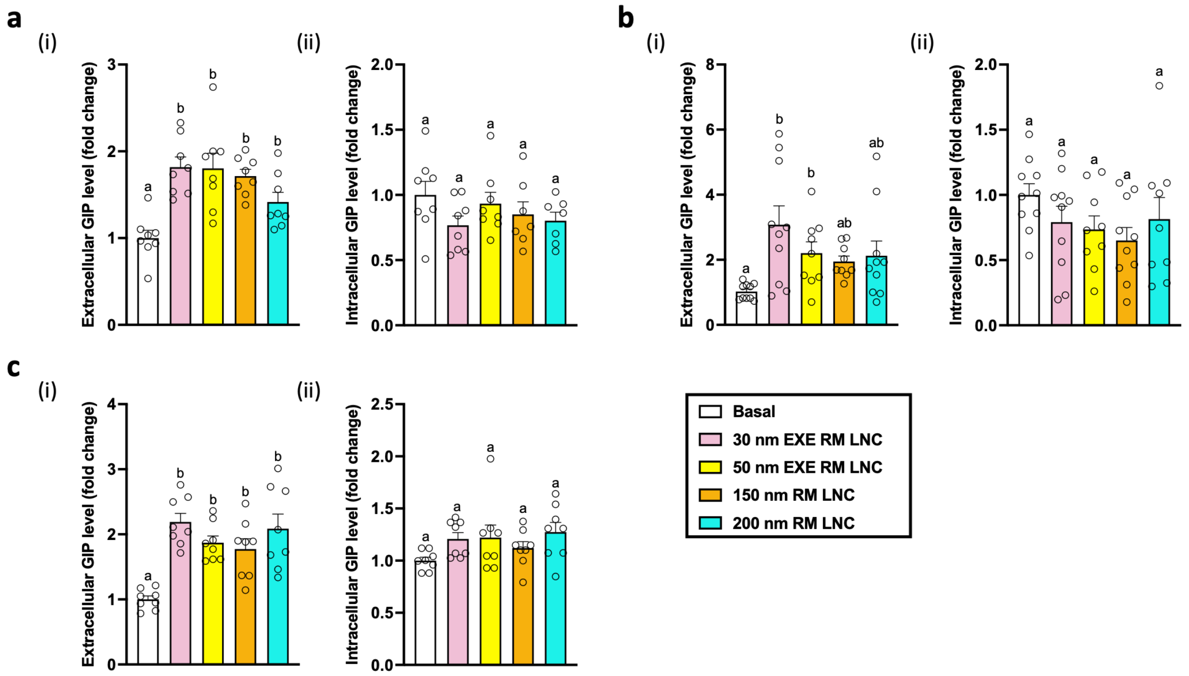

Fig. S7: RM LNC with different particle size stimulate GIP secretion in mouse and human intestinal organoids *ex vivo*. a-c, Total GIP level in supernatants (i) and lysates (ii) measured by ELISA after 2 h of coincubation with RM LNC of different sizes (from 30 nm to 200 nm) in 2D monolayer of mouse GIP crextdRFP duodenal organoids (**a**), mouse jejunal organoids (**b**) and human GIP Venus duodenal organoids (**c**). The tested nanoparticles are dispersed in 138 buffer containing 1 mM glucose (Basal buffer) at concentration of 2 mg/ml. All data are expressed as fold change of total GIP level in organoids responding to basal buffer. Data with different superscript letters are significantly different ($p<0.05$), determined by a one-way ANOVA followed by Tukey's post hoc test. The data are presented as mean ± SEM (n=7-10), corresponding to 4-5 independent experiments.

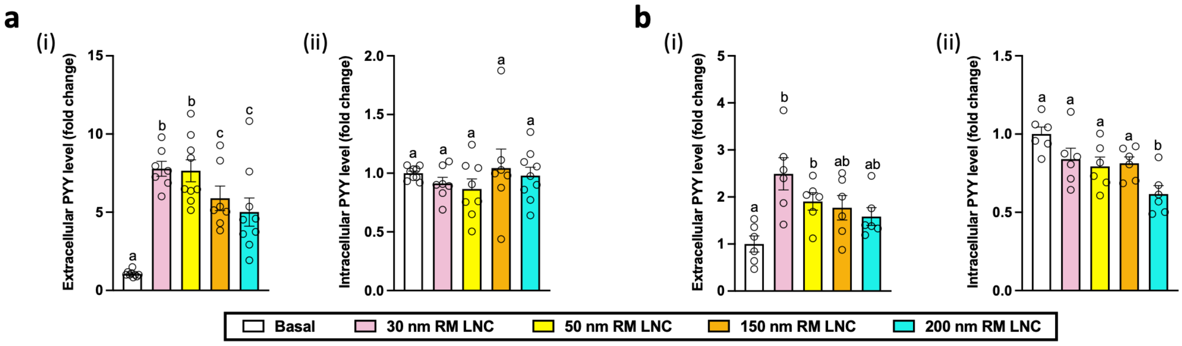

Fig. S8: RM LNC with different particle size stimulate PYY secretion in mouse and human intestinal organoids *ex vivo*. a,b, Total PYY level in supernatants (i) and lysates (ii) measured by ELISA after 2 h of coincubation with RM LNC of different sizes (from 30 nm to 200 nm) in 2D monolayer of mouse GLU EPAC2-GFP ileal organoids (**a**) and human GLU-Venus ileal organoids (**b**). The tested nanoparticles were dispersed in 138 buffer containing 1 mM glucose (Basal buffer) at concentration of 2 mg/ml. All data are expressed as fold change of total PYY level in organoids responding to basal buffer. Data with different superscript letters are significantly different ($p<0.05$), determined by a one-way ANOVA followed by Tukey's post hoc test. The data are presented as mean ± SEM (n=6-10), corresponding to 3-4 independent experiments.

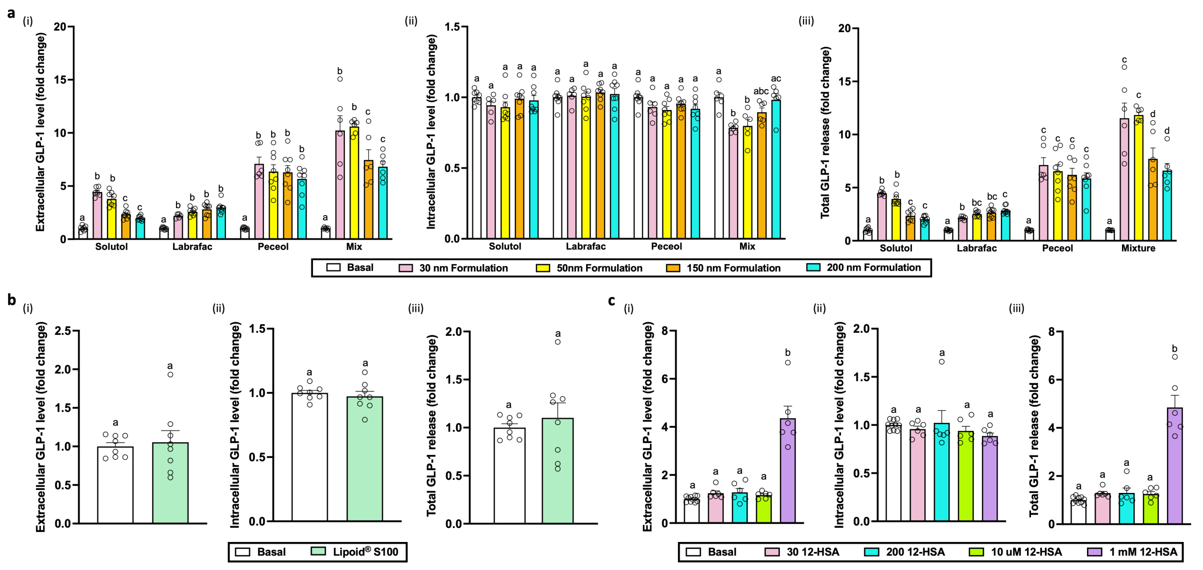

Fig. S9: Main excipients in RM LNC mediate GLP-1 secretion in intestinal organoids *ex vivo*. a, GLP-1 secretion from mouse GLU EPAC2-GFP ileal organoids in 2D monolayer, on addition of different concentration of Solutol® HS 15, Labrafac® lipophile WL 1349, Peceol® and the mixtures of three main components in the presence of 138 buffer containing 1 mM glucose (basal buffer). The amount of each component corresponds to that contained in RM LNC of different sizes (30 nm, 50 nm, 150 nm and 200 nm). Data are presented as mean ± SEM (n=6-8), corresponding to 3-4 independent experiments. P values were determined by a one-way ANOVA followed by Tukey's post hoc test. **b,** GLP-1 release from mouse GLU EPAC2-GFP ileal organoids in 2D monolayer in response to 23.37 nM of Lipoid® S100 in the presence of basal buffer (mean ± SEM) (n=7-8, N=3). P values were analyzed by the Mann-Whitney test. **c,** GLP-1 release from L cells in mouse GLU EPAC2-GFP ileal organoids in 2D monolayer after incubation with various concentrations of 12-hydroxystearic acid (including 0.122 µM, 0.687 µM, 10 µM and 1 mM) (mean ± SEM, n=6-10, N=3). P values were determined by a one-way ANOVA followed by Tukey's post hoc test. All data (i-ii) are expressed as fold change of total GLP-1 level in organoids responding to basal buffer. Total GLP-1 release (iii) was calculated by the correction of total GLP-1 level quantified in supernatants (i) and lysates (ii) after incubation for 2h by ELISA. Data with different superscript letters are significantly different ($p<0.05$).

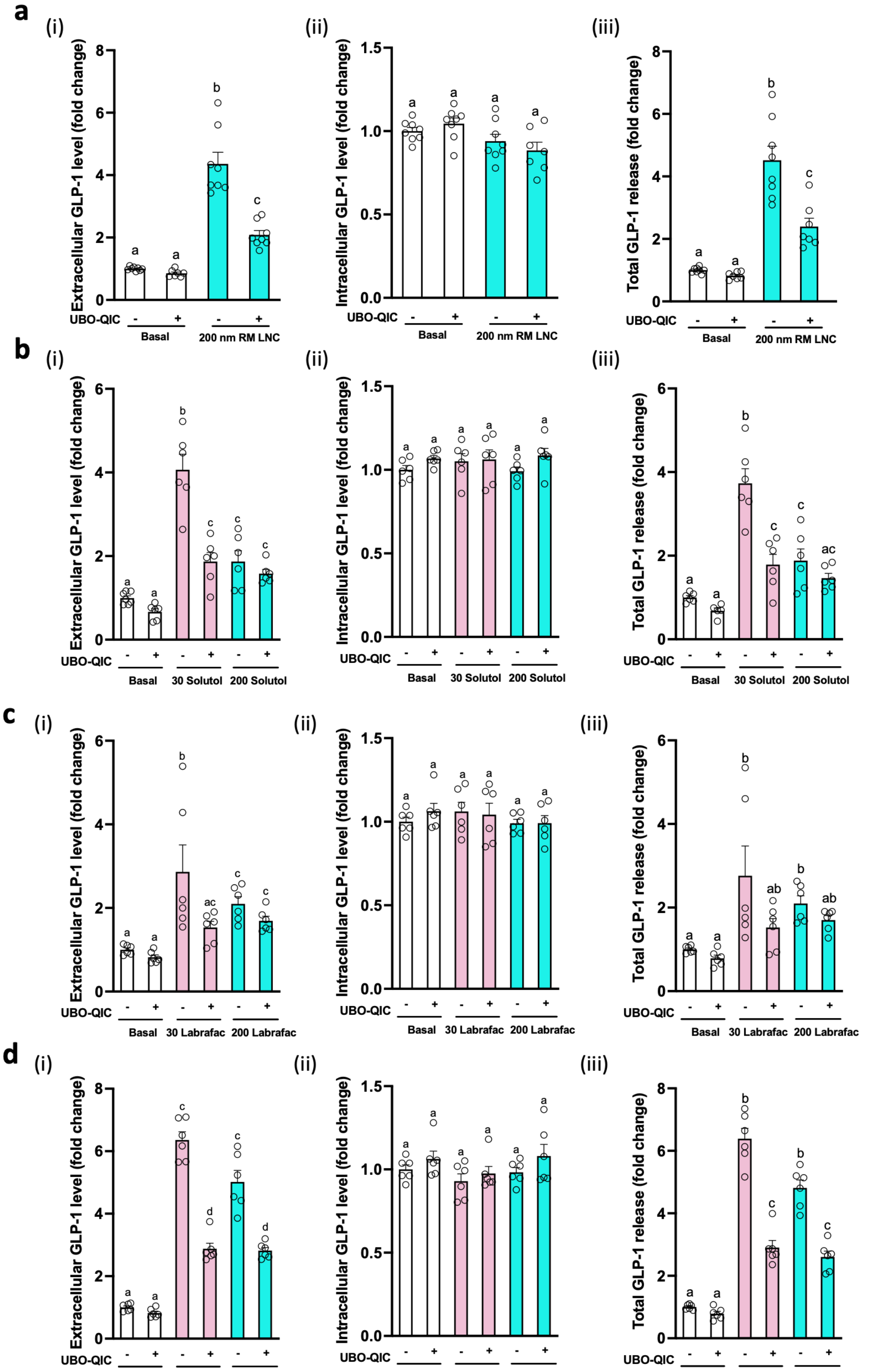

Fig. S10: The role of Gq-coupled receptor in RM-LNC and its main excipients on GLP-1 secretion from enteroendocrine L cells *ex vivo*. a-d, GLP-1 secretion from the 2D monolayer of mouse GLU EPAC2-GFP ileal organoids after 2h incubation of RM LNC (**a**), Solutol® HS 15 (**b**), Labrafac® lipophile WL 1349 (**c**) and Peceol® (**d**) with or without the presence of UBO-QIC (1 μM). All data (i-ii) are expressed as fold change of total GLP-1 level in organoids responding to basal buffer. Total GLP-1 release (iii) is analyzed by total GLP-1 level measured in supernatants (i) and lysates (ii) by ELISA. For all figures, data are shown as mean ± SEM (n=6-8) of 3-4 independent experiments. Statistical comparison (different superscript letters; $p<0.05$) is performed by one-way ANOVA followed by Tukey's post hoc test.

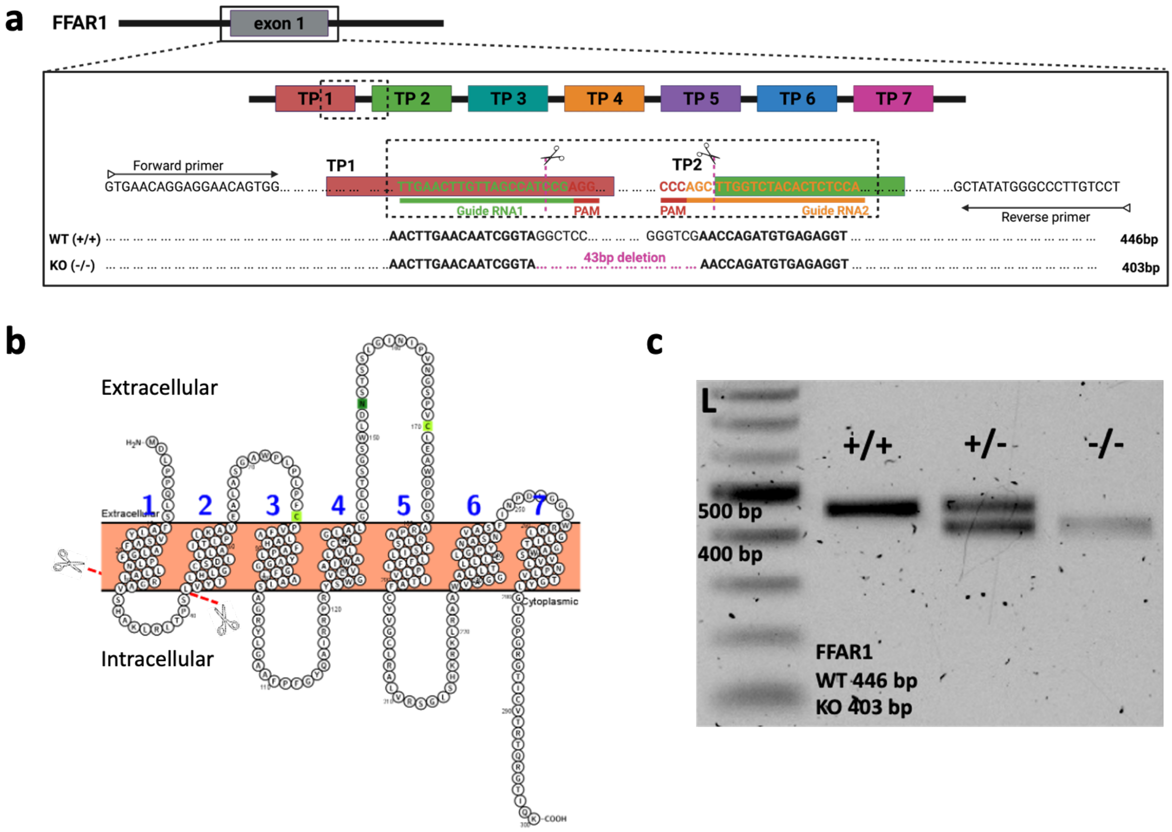

Fig. S11: CRISPR/Cas9–mediated genome editing uses a dual short guide RNA strategy to knockout *Ffar1* gene in cas9-GFP mouse ileal organoids. a, A schematic drawing shows the strategy to generate FFAR1 KO mouse ileal organoids. Two sgRNAs were designed to delete 43 bp between the nucleotide sequences of transmembrane helices 1 and 2 from exon 1 in the *Ffar1* gene. The binding sites for guide RNAs are highlighted with the protospacer adjacent motif (PAM) sequence in red, and the cutting sites are indicated by scissors**.** The nucleotide sequences of wild-type (WT) and KO alleles are shown. **b,** Topological structure of FFAR1 was generated using Protter. The deleted amino acid sequences are marked by scissors. **c,** A representative PCR genotyping result for WT (+/+), heterozygous (+/-) and homozygous (-/-) KO mouse ileal organoids. Genomic DNA was extracted from picked organoids of different genotypes and subjected to amplification by PCR. A forward primer that anneals upstream of transmembrane helix 1 and a reverse primer that anneals downstream of transmembrane helix 2 shows a successful deletion of 43 bp. The deduced genotype is shown the agarose gel. L, size standards.

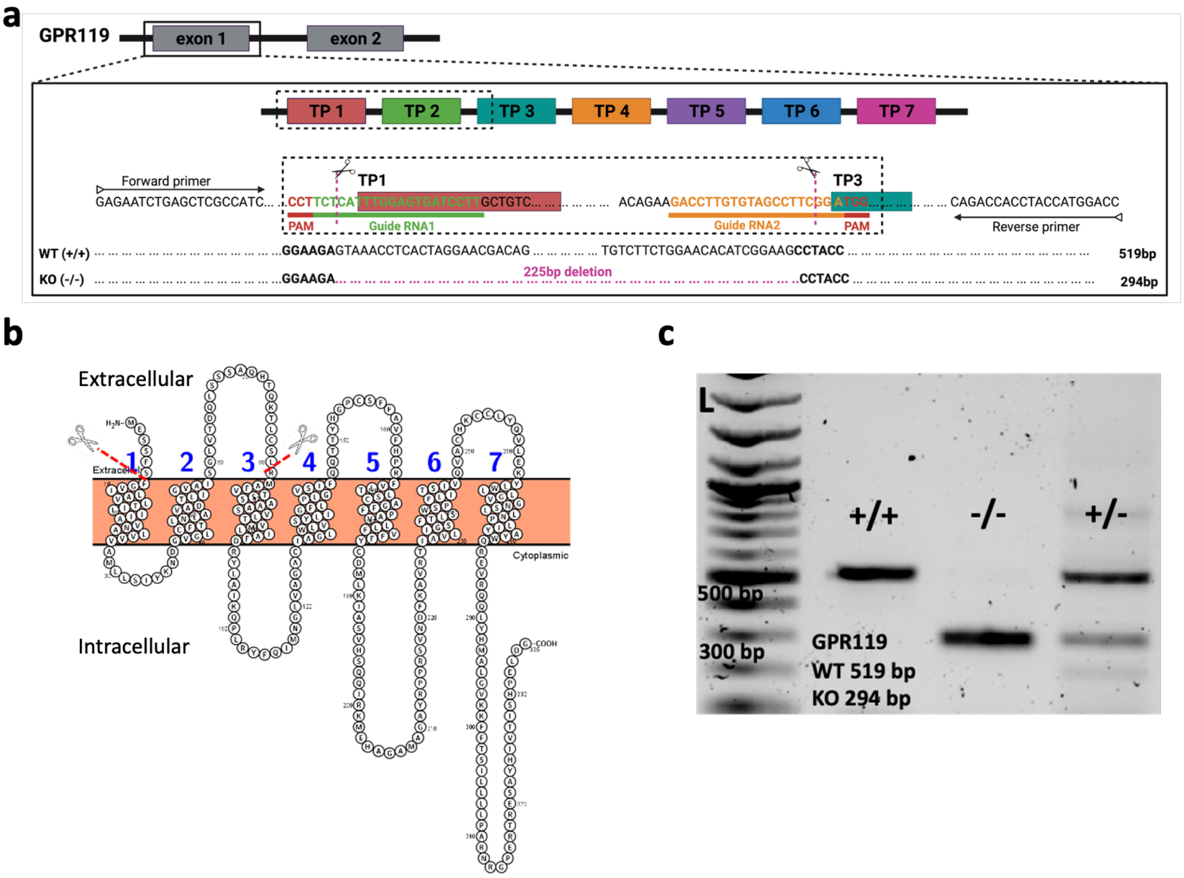

Fig. S12: CRISPR/Cas9–mediated genome editing uses a dual short guide RNA strategy to knockout *Gpr119* gene in cas9-GFP mouse ileal organoids. a, A schematic drawing shows the strategy to generate GPR119 KO mouse ileal organoids. Two sgRNAs were designed to delete 225 bp between the nucleotide sequences of transmembrane helices 1 and 3 from exon 1 in *Gpr119*. The binding sites for guide RNAs are highlighted with the PAM sequence in red, and the cutting sites are indicated by scissors**.** The nucleotide sequences of WT and KO alleles are shown. **b,** Topological structure of GPR119 was generated using Protter. The deleted amino acid sequences are marked by scissors. **c,** A representative PCR genotyping result for WT (+/+), homozygous (-/-) KO and heterozygous (+/-) mouse ileal organoids. Genomic DNA was extracted from picked organoids of different genotypes and subjected to amplification by PCR. A forward primer that anneals upstream of transmembrane helix 1 and a reverse primer that anneals downstream of transmembrane helix 3 shows a successful deletion of 225 bp. The deduced genotype is shown the agarose gel. L, size standards.

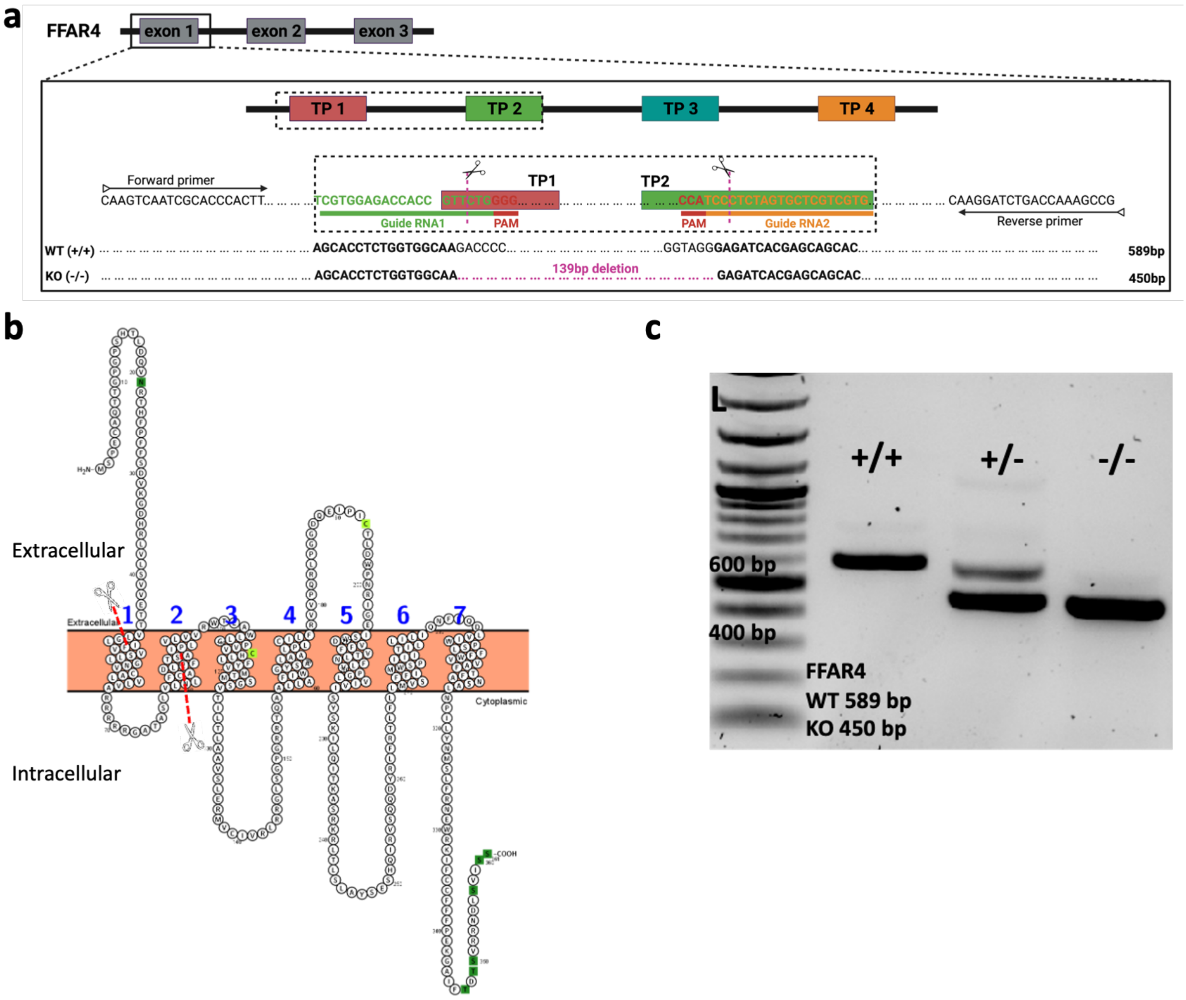

Fig. S13: CRISPR/Cas9–mediated genome editing uses a dual short guide RNA strategy to knockout *Ffar4* gene in cas9-GFP mouse ileal organoids. a, A schematic drawing shows the strategy to generate FFAR4 KO mouse ileal organoids. Two sgRNAs were designed to delete 139 bp between the nucleotide sequences of transmembrane helices 1 and 2 from exon 1 in *Ffar4*. The binding sites for guide RNA are highlighted with PAM sequence in red, and the cutting sites are indicated by scissors**.** The nucleotide sequences of WT and KO alleles are shown. **b,** Topological structure of FFAR4 was generated using Protter. The deleted amino acid sequences are marked by scissors. **c,** A representative PCR genotyping result for WT (+/+), heterozygous (+/-), and homozygous (-/-) KO mouse ileal organoids. Genomic DNA was extracted from picked organoids of different genotypes and subjected to amplification by PCR. A forward primer that anneals upstream of transmembrane helix 1 and a reverse primer that anneals downstream of transmembrane helix 2 shows a successful deletion of 139 bp. The deduced genotype is shown the agarose gel. L, size standards.

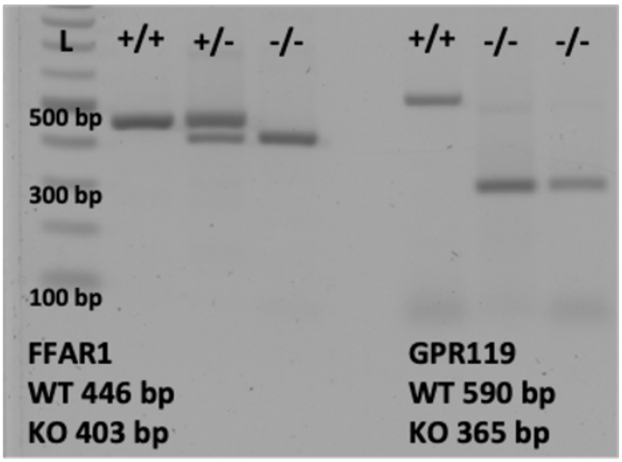

Fig. S14: CRISPR/Cas9–mediated genome editing uses a dual short guide RNA strategy to knockout *Ffar1* and *Gpr119* gene in cas9-GFP mouse ileal organoids. A representative PCR genotyping result for *Ffar1* WT (+/+), heterozygous (+/-) and homozygous (-/-) KO in mouse GPR119 KO ileal organoids. The deduced genotype is shown on the agarose gel. L, size standards.

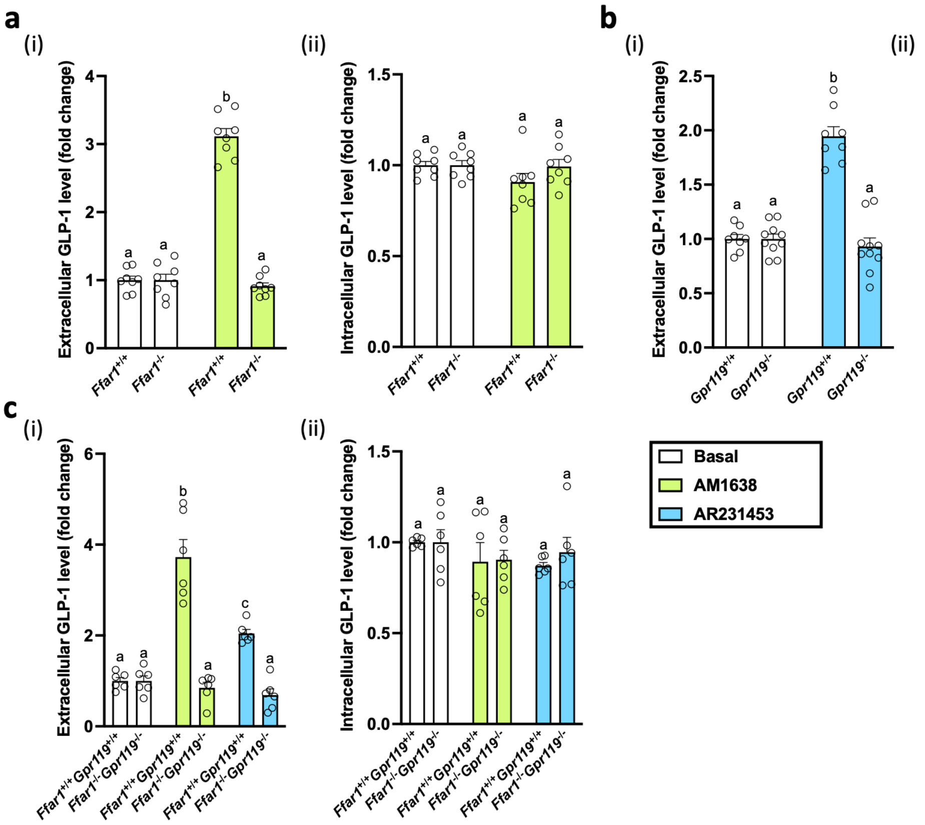

Fig. S15: Different G-coupled protein receptor agonists induce GLP-1 release in mouse ileal WT and KO organoids. a-c, Total GLP-1 level in supernatants (i) and lysates (ii) measured by ELISA after 2 h of incubation with FFAR1 agonist AM1638 (10 μM) or GPR119 agonist AR231453 (0.1 μM) in 2D monolayers of cas9-GFP mouse ileal WT organoids and *Ffar1*$^{-/-}$ organoids (**a**), *Gpr119*$^{-/-}$ organoids (**b**) and *Ffar1*$^{-/-}$*Gpr*119$^{-/-}$ organoids (**c**). All data are expressed as fold change of total GLP-1 level in organoids measured in parallel in basal buffer. The data are presented as mean ± SEM (n=6-10), corresponding to 3-5 independent experiments. Data with different superscript letters are significantly different ($p<0.05$), determined by a one-way ANOVA followed by Tukey's post hoc test.

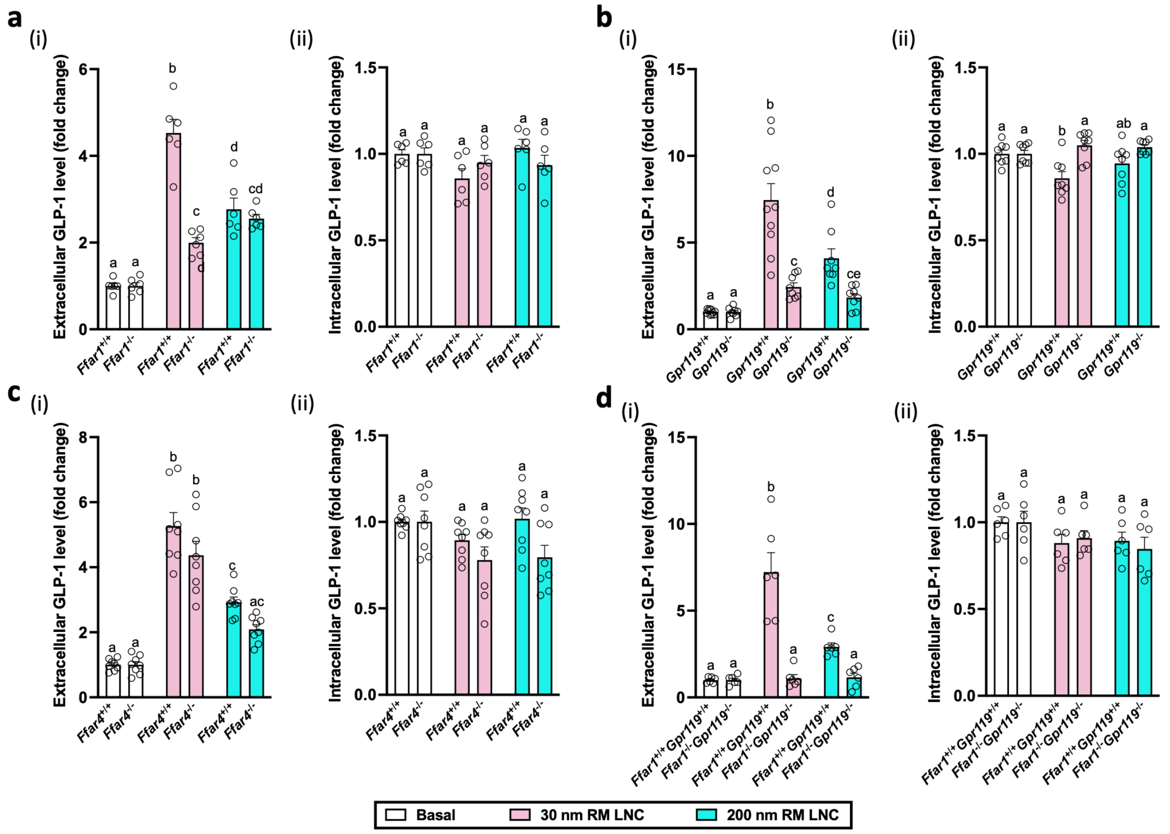

Fig. S16: 30 nm and 200 nm RM LNC stimulate GLP-1 secretion in mouse ileal WT and KO organoids. a-d, Total GLP-1 levels in supernatants (i) and lysates (ii) measured by ELISA after 2 h of incubation with 2 mg/ml RM LNC (particle size: 30 nm or 200 nm) in 2D monolayers of cas9-GFP mouse ileal WT organoids and *Ffar1*$^{-/-}$ (**a**), *Gpr119*$^{-/-}$ (**b**), *Ffar4*$^{-/-}$ (**c**) and *Ffar1*$^{-/-}$*Gpr119*$^{-/-}$(**d**) organoids. All data are expressed as fold change of total GLP-1 level in organoids compared with that measured in parallel in basal buffer (mean ± SEM, n=6-8, N=3-4). Data with different superscript letters are significantly different ($p<0.05$), determined by a one-way ANOVA followed by Tukey's post hoc test.

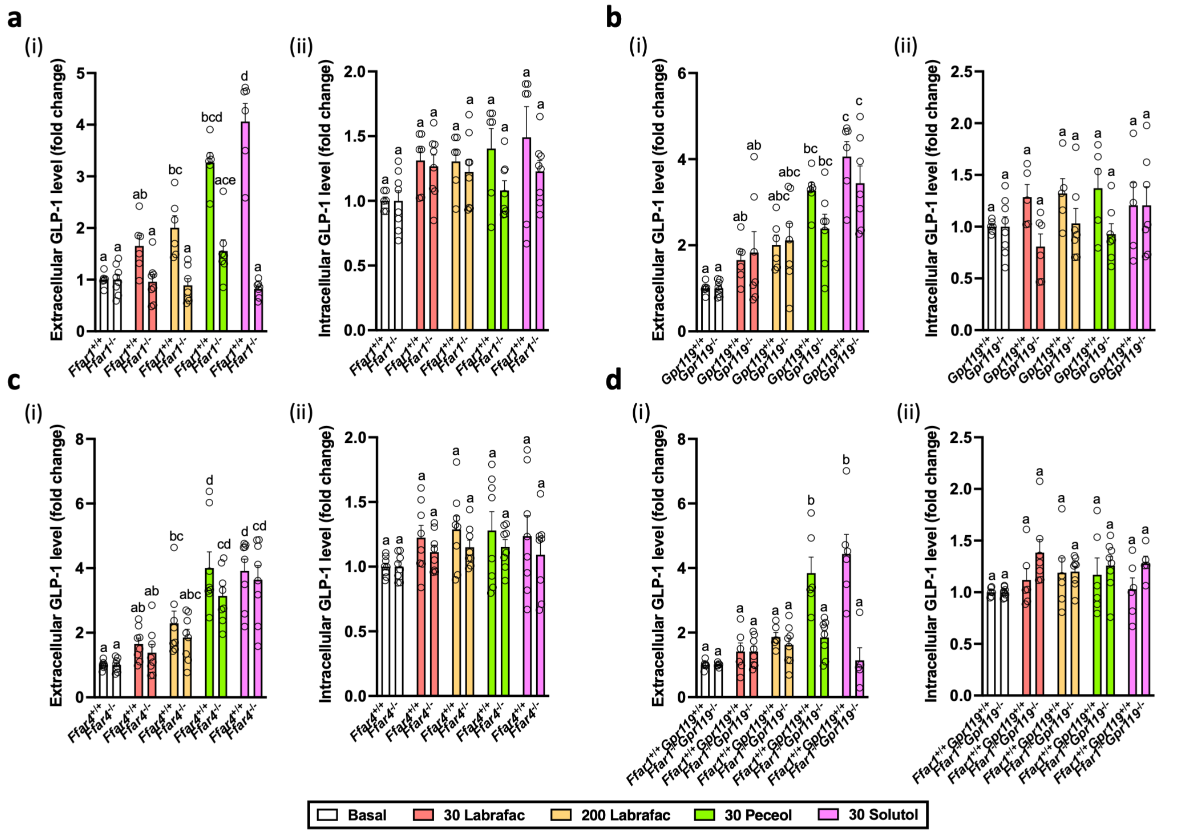

Fig. S17: Excipients in RM LNC trigger GLP-1 release in mouse ileal WT and KO organoids. a-d, Total GLP-1 level in supernatants (i) and lysates (ii) measured by ELISA after 2 h of incubation with 0.651 µM Labrafac® lipophile WL 1349 (30 Labrafac), 3.598 µM Labrafac® lipophile WL 1349 (200 Labrafac), 0.666 µM Peceol® (30 Peceol) and 0.981 µM Solutol® HS 15 (30 Solutol) in 2D monolayers of cas9-GFP mouse ileal WT organoids and *Ffar1*$^{-/-}$ (**a**), *Gpr119*$^{-/-}$ (**b**), *Ffar4*$^{-/-}$ (**c**) and *Ffar1*$^{-/-}$*Gpr119*$^{-/-}$ (**d**) organoids. All data are expressed as fold change of total GLP-1 level in organoids compared with that measured in parallel in basal buffer (mean ± SEM, n=5-8, N=3-4). Data with different superscript letters are significantly different ($p<0.05$), determined by a one-way ANOVA followed by Tukey's post hoc test.

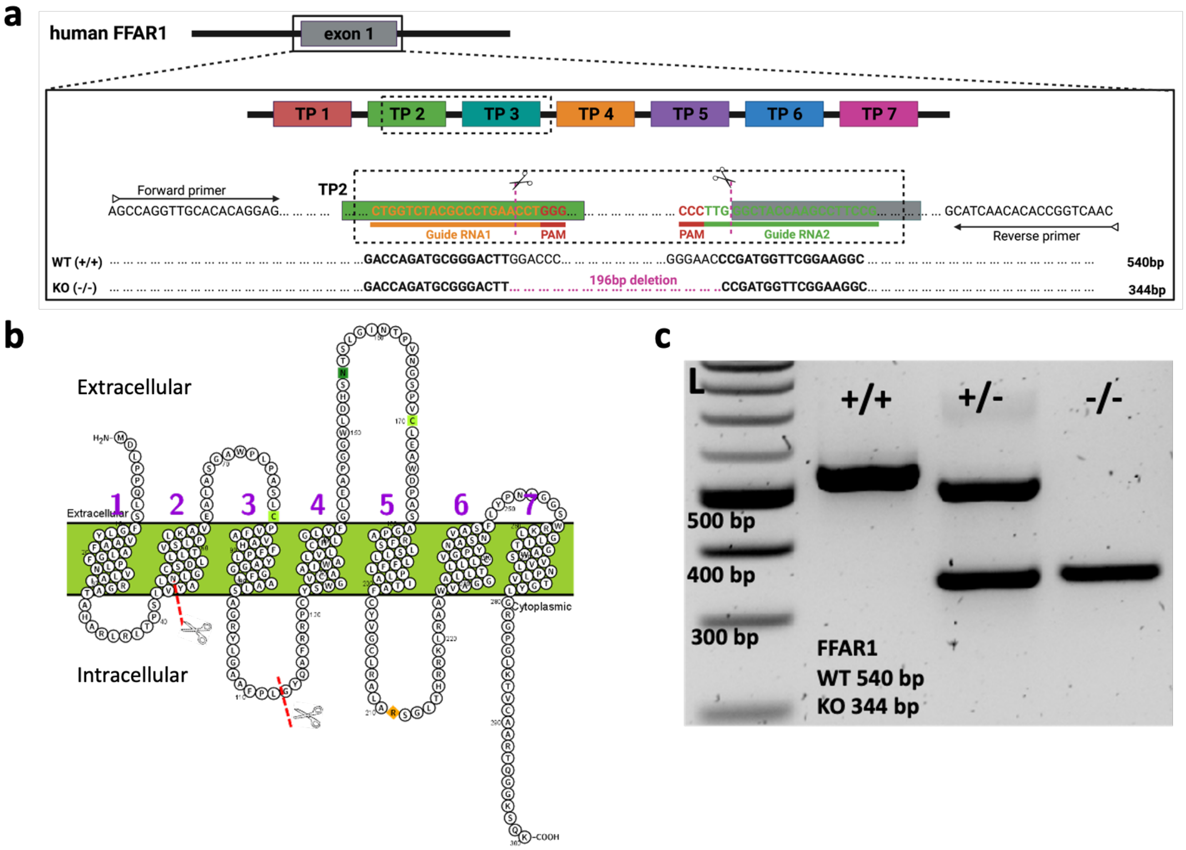

Fig. S18: CRISPR/Cas9–mediated genome editing uses two sgRNA plasmid strategy to knockout *FFAR1* gene in human GLU-Venus ileal organoids. a, A schematic drawing shows the strategy to generate GPR40 KO human ileal organoids. Two sgRNA plasmid are designed to delete 196 bp between the nucleotide sequences of transmembrane helices 2 and 4 from exon 1 in the *FFAR1* gene. The binding sites for guide RNAs are highlighted with PAM sequences in red, and cutting sites indicated by scissors**.** The nucleotide sequences of WT and KO alleles are shown. **b,** Topological structure of FFAR1 was generated using Protter. The deleted amino acid sequences are marked by scissors. **c,** A representative PCR genotyping result for WT (+/+), heterozygous (+/-) and homozygous (-/-) KO human ileal organoids. Genomic DNA was extracted from picked organoids of different genotypes and subjected to amplification by PCR. A forward primer that anneals upstream of the sequence for transmembrane helix 1 and a reverse primer that anneals downstream of the sequence for transmembrane helix 4 shows a successful deletion of 196 bp. The deduced genotype is shown on the agarose gel. L, size standards.

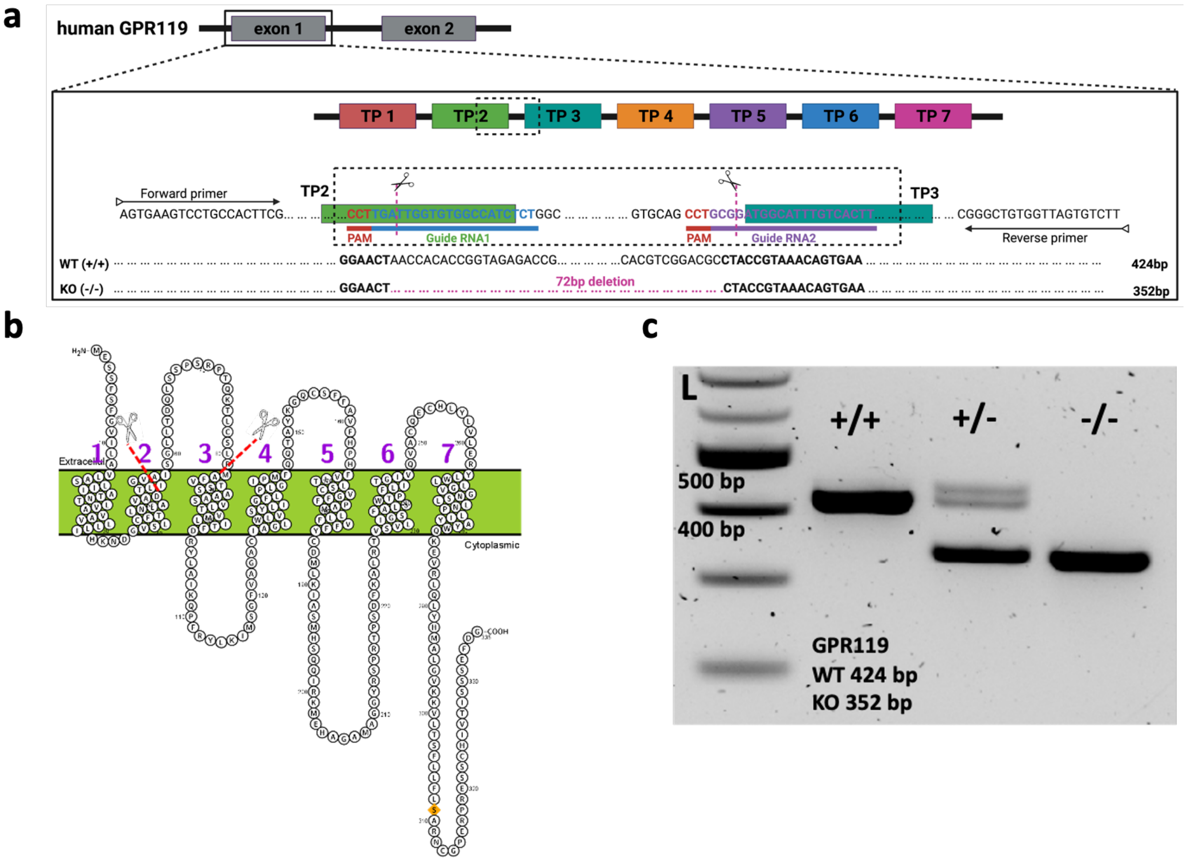

Fig. S19: CRISPR/Cas9–mediated genome editing uses two sgRNA plasmid strategy to knockout *GPR119* gene in human GLU-Venus ileal organoids. a, A schematic drawing shows the strategy to generate GPR119 KO human ileal organoids. Two sgRNA plasmid are designed to delete 72 bp between the nucleotide sequences of transmembrane helices 2 and 3 from exon 1 of *GPR119* gene. The binding sites for guide RNAs are highlighted, with PAM sequences in red, and the cutting sites indicated by scissors**.** The nucleotide sequences of WT and KO alleles are shown. **b,** Topological structure of GPR119 was generated using Protter. The deleted amino acid sequences are marked by scissors. **c,** A representative PCR genotyping result for WT (+/+), heterozygous (+/-) and homozygous (-/-) KO human ileal organoids. Genomic DNA was extracted from picked organoids of different genotypes and subjected to amplification by PCR. A forward primer that anneals upstream of the sequence encoding transmembrane helix 2 and a reverse primer that anneals downstream of the sequence encoding transmembrane helix 3 shows a successful deletion of 72 bp. The deduced genotype is shown on the agarose gel. L, size standards.

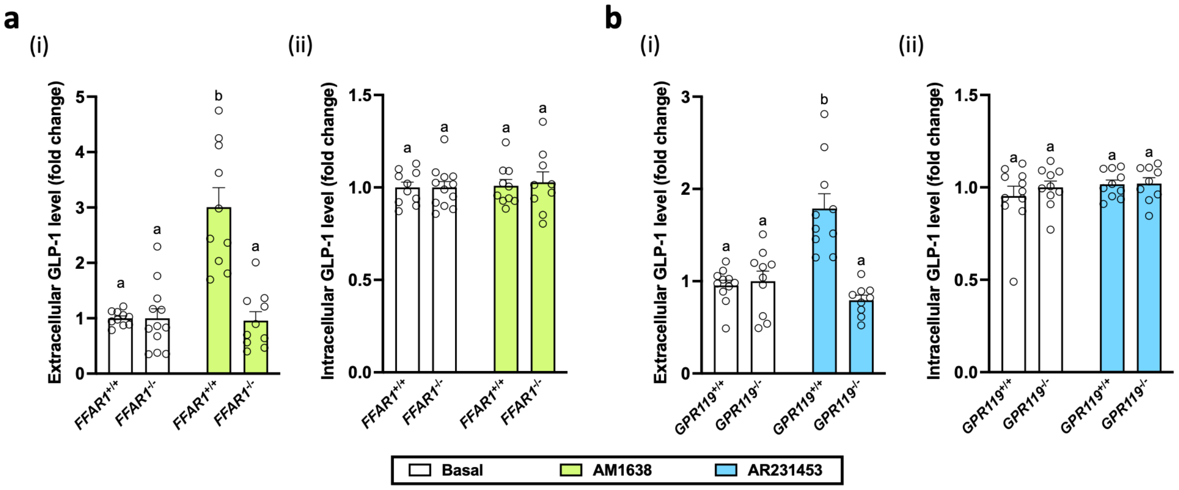

Fig. S20: Different G-coupled protein receptor agonists induce GLP-1 release in human ileal WT and KO organoids. a,b, Total GLP-1 level in supernatants (i) and lysates (ii) measured by ELISA after 2 h incubation with the FFAR1 agonist AM1638 (10 µM) or the GPR119 agonist AR231453 (0.1 µM) in 2D monolayers of GLU-Venus human ileal WT organoids and *FFAR1*$^{-/-}$ organoids (**a**) and *GPR119* $^{-/-}$ organoids (**b**). All data are expressed as fold change of total GLP-1 level in organoids compared with that measured in parallel in basal buffer. The data are presented as mean ± SEM (n=8-12), corresponding to 4-5 independent experiments. Data with different superscript letters are significantly different ($p<0.05$), determined by a one-way ANOVA followed by Tukey's post hoc test.

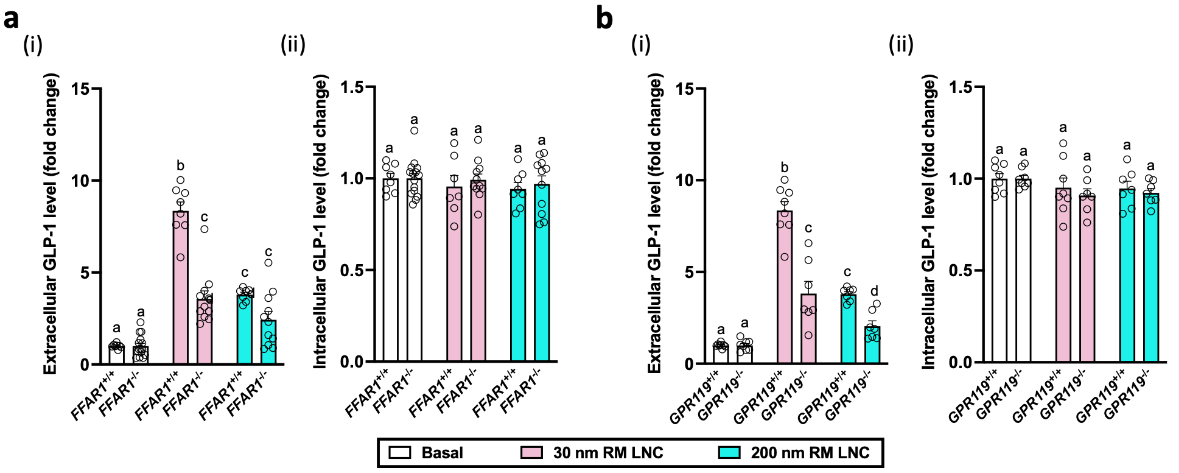

Fig. S21: 30 nm and 200 nm RM LNC stimulate GLP-1 secretion in human ileal WT and KO organoids. a-d, Total GLP-1 level in supernatants (i) and lysates (ii) measured by ELISA after 2 h incubation with 2 mg/ml RM LNC (particle size: 30 nm or 200 nm) in 2D monolayers of GLU-Venus human ileal WT organoids and *FFAR1*$^{-/-}$ (**a**) and *GPR119*$^{-/-}$ (**b**). All data are expressed as fold change of total GLP-1 level in organoids compared with that measured in parallel in basal buffer (mean ± SEM, n=7-15, N=3-5). Data with different superscript letters are significantly different ($p<0.05$), determined by a one-way ANOVA followed by Tukey's post hoc test.

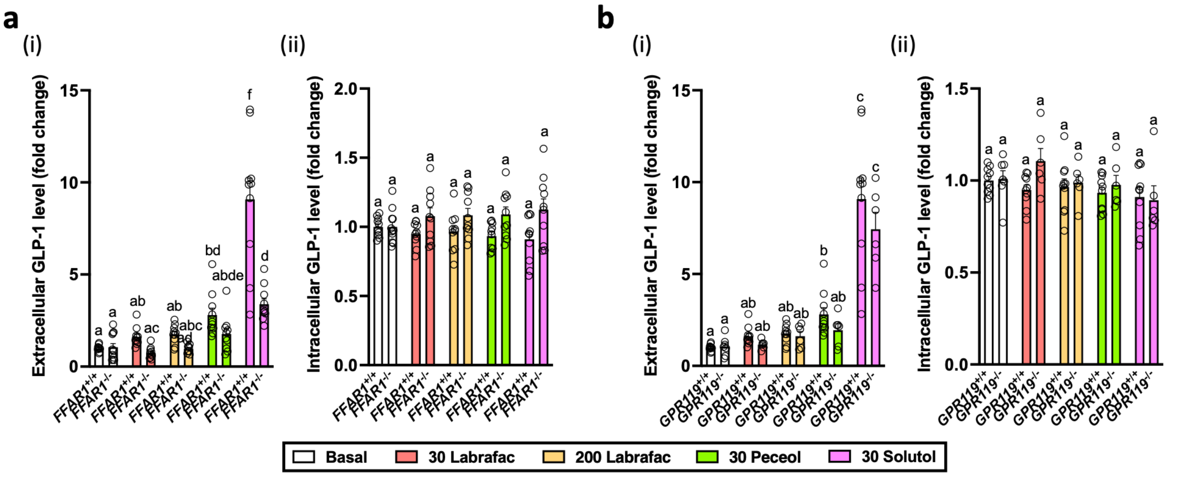

Fig. S22: Excipients in RM LNC trigger GLP-1 release in human ileal WT and KO organoids. a-d, Total GLP-1 level in supernatants (i) and lysates (ii) measured by ELISA after 2 h incubation with 0.651 µM Labrafac® lipophile WL 1349 (30 Labrafac), 3.598 µM Labrafac® lipophile WL 1349 (200 Labrafac), 0.666 µM Peceol® (30 Peceol) and 0.981 µM Solutol® HS 15 (30 Solutol) in 2D monolayer of GLU-Venus human ileal WT organoids and *FFAR1*$^{-/-}$ (**a**) and *GPR119*$^{-/-}$ (**b**) organoids. All data are expressed as fold change of total GLP-1 level in organoids compared with that measured in parallel in basal buffer (mean ± SEM, n=6-12, N=3-5). Data with different superscript letters are significantly different ($p<0.05$), determined by a one-way ANOVA followed by Tukey's post hoc test.

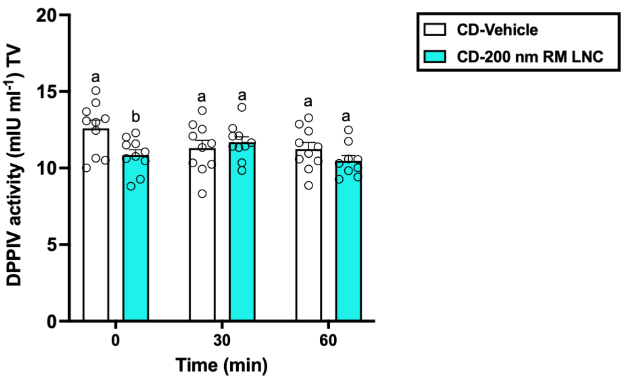

Fig. S23: Effect of lipid-based nanocarriers on dipeptidyl peptidase-4 activity *in vivo*. Dipeptidyl peptidase-4 activities were measured in plasma from tail vein before, 30 min and 60 min after oral gavage of 200 nm RM LNC (1.62 mg/g lipid dose) in normoglycemic mice (n=9-10). Data are presented as the mean ± SEM, and were determined by a one-way ANOVA followed by Tukey's post hoc test. Data with different superscript letters are significantly different ($p<0.05$).

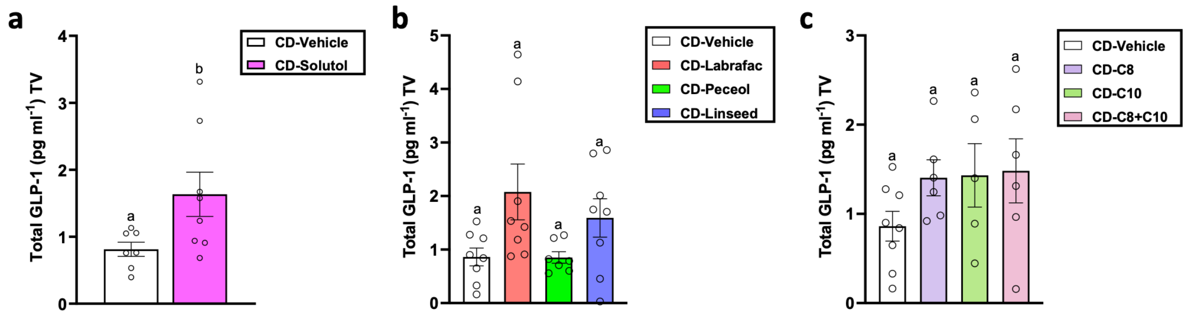

Fig. S24: Secretory effect of excipients and fatty acids *in vivo*. a, Total GLP-1 levels were measured in mouse plasma from tail vein at 60 min post oral gavage of MilliQ water (vehicle) and Solutol® (0.83 mM/kg) (mean ± SEM, n=6-8). **b**, Total GLP-1 levels were tested from tail vein after 60 min of oral administration of vehicle (DMSO: Tween 80: MilliQ water in 1: 1: 19 ratio), Labrafac® lipophile WL 1349 (4.32 mM/kg), Peceol® (0.56 mM/kg) and linseed oil (4.44 mM/kg). Data were presented as mean ± SEM (n=6-8). **c**, Plasma total GLP-1 levels were tested from tail vein post 60 min of oral administration of vehicle (DMSO: Tween 80: MilliQ water in 1: 1: 19 ratio), caprylic acid (C8; 2.16 mM/kg), capric acid (C10; 2.16 mM/kg) and their mixtures (C8+C10). Data shown are mean ± SEM (n=6-8). All data were determined by a one-way ANOVA followed by Tukey's post hoc test. Data with different superscript letters are significantly different ($p<0.05$).

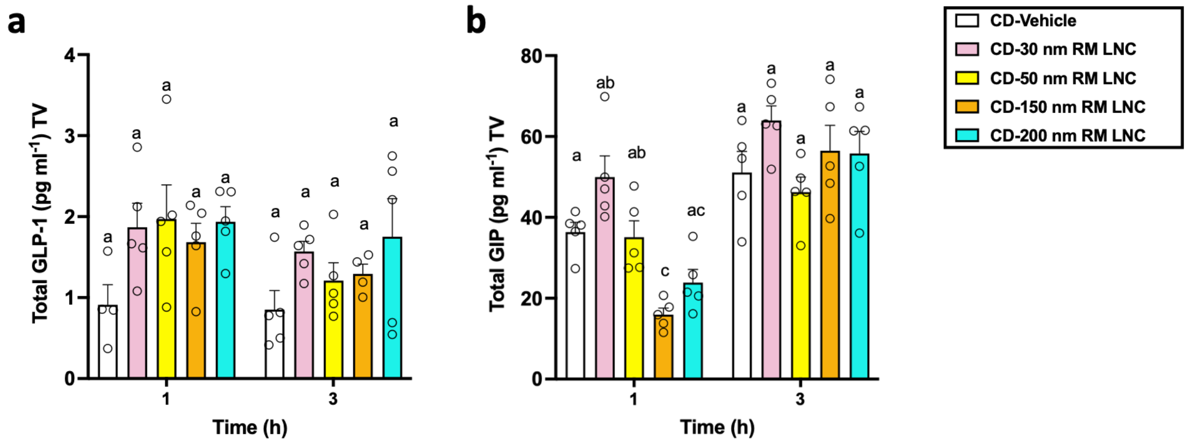

Fig. S25: Size effect of RM LNC-mediated incretin hormone release in mice. Total GLP-1 (**a**) and total GIP levels (**b**) in mouse plasma from tail vein 1 and 3 hours after oral administration of MilliQ water (Vehicle) and reverse micelle lipid nanocapsules (RM LNC) with different particle sizes (including 30 nm, 50 nm, 150 nm and 200 nm). Data shown are mean ± SEM. n = 4 or 5 mice per group. Data with different superscript letters are significantly different ($p<0.05$) according to two-way analysis of variance followed by Tukey's post hoc test or Mann-Whitney test.

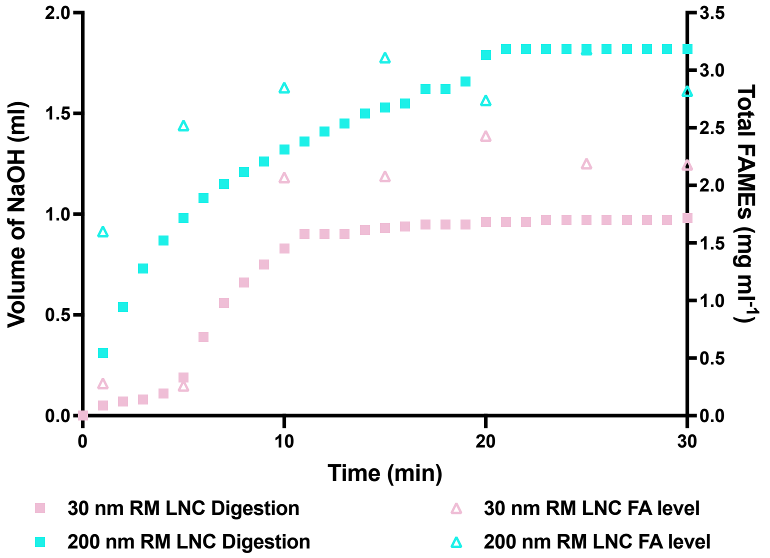

Fig. S26: Digestion of lipid nanocarriers differing in size/composition. Digestion profiles of lipid-based formulations (30 nm RM LNC and 200 nm RM LNC) and corresponding amounts of fatty acids generated during digestion. Pancreatic lipase was added at time = 0 min to initiate digestion and the drop in pH was titrated with NaOH solution to maintain the pH at 6.5 (representative of small intestine). Aliquots were removed for fatty acid analysis by gas chromatography (FAEMs: fatty acid methyl esters).

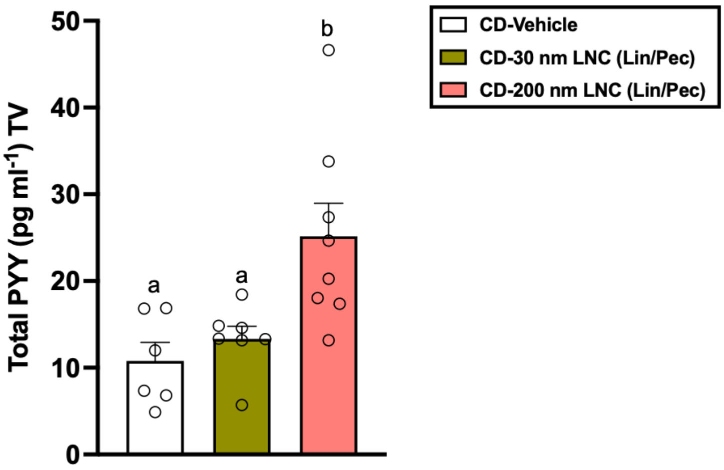

Fig. S27: Effect of linseed oil-based nanocarriers on PYY secretion *in vivo*. Total PYY levels were measured in mouse plasma from tail vein at 60 min post oral gavage of MilliQ water (vehicle) or empty lipid-based nanocarriers containing linseed oil (Lin/Pec), including 30 nm RM LNC (Lin/Pec) and 200 nm RM LNC (Lin/Pec) (1.62 mg/g lipid dose). Data shown are mean ± SEM (n = 6-7 mice per group). Data with different superscript letters are significantly different ($p<0.05$) according to one-way ANOVA followed by Tukey's post hoc test.

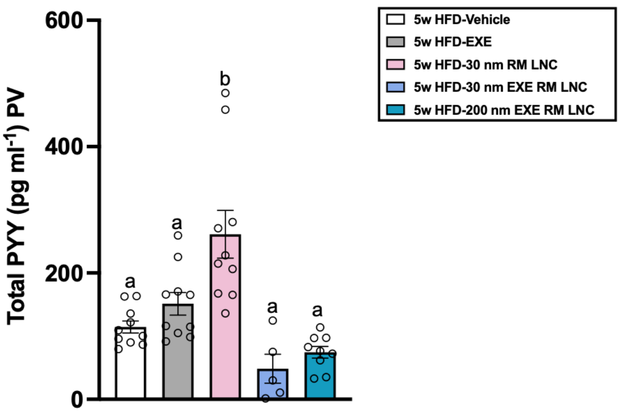

Fig. S28: Effect of RM LNC on PYY secretion *in vivo*. Total PYY levels were measured in mouse plasma from the portal vein at 60 min post oral gavage of MilliQ water (vehicle), exenatide in solution (EXE), empty lipid-based nanocarriers (30 nm RM LNC), and exenatide-loaded lipid-based nanocarriers (30 nm EXE RM LNC and 200 nm RM LNC) (1.62 mg/g in lipid dose, 500 µg/kg in drug dose). Data shown are mean ± SEM (n = 5-10 mice per group). Data with different superscript letters are significantly different (p<0.05) according to one-way ANOVA followed by Tukey's post hoc test.

Table S1: Composition of RM LNC with different particle sizes.

LNCs Size (nm)	30	50	150	200
**Reverse Micelles (RM)**				
Drug Solution (µL)	50	50	50	50
Span 80® (mg)	100	100	100	100
Labrafac® (mg)	500	500	500	500
**Lipid nanocapsules (LNC)**				
Solutol® HS15 (mg)	700	400	220	120
Lipoid® S100 (mg)	13.4	13.4	13.4	13.4
Labrafac® (mg)	144	219.8	761.5	769.5
Peceol® (mg)	176	146.2	150.5	85.5
NaCl (mg)	50	50	50	50
MilliQ Water (µL)	980	1234	868	1025
MilliQ Water at 0 °C (µL)	2500	2500	2500	2500
Temperature Cycle (°C)	45-60	45-60	45-60	50-65
PIZ (°C)	50-53	50-52	50-52	59-61

Table S2: Physicochemical characterization of RM-LNC presenting different sizes (mean ± SEM, n=3).

LNCs Size (nm)	30	50	150	200
Size (nm)	35.0±1.4	54.9±1.3	149.6±1.5	224.6±1.1
PDI	0.03±0.01	0.04±0.01	0.06±0.01	0.14±0.02
Zeta (mV)	-2.04±0.74	-1.54±0.22	-3.07±0.38	-3.83±0.32

Table S3: The concentration of main components in RM LNC presenting different sizes (e*x vivo* tested NPs concentration: 2 mg/ml).

Formulations	Solutol® (µM)	Labrafac® (µM)	Peceol® (µM)	Lipoid® S100 (nM)
30 nm RM LNC	0.981	0.651	0.666	23.37
50 nm RM LNC	0.681	1.207	0.674	23.37
150 nm RM LNC	0.291	3.246	0.537	23.37
200 nm RM LNC	0.174	3.598	0.335	23.37

Table S4: The concentration of 12-hydroxystearic acid (12-HSA) in the Solutol® HS 15 contained in RM LNC presenting different sizes (e*x vivo* tested concentration NPs concentration: 2 mg/ml).

Counting 70 % of solutol with NPs concentration 2 mg/ml	
**RM-LNCs Size (nm)**	**12-hydroxy stearic acid (µM)**
30	0. 687
200	0. 122

Table S5: The sequence of guide RNAs used in mouse organoids.

Targeting gene	gRNA	Strand	Sequence	PAM
Ffar1	1	-	TTGAACTTGTTAGCCATCCG	AGG
	2	+	TGGAGAGTGTAGACCAAGCT	GGG
Gpr119	1	+	AAGGATCACTCCAAATGAGA	AGG
	2	-	GACCTTGTGTAGCCTTCGGA	TGG
Ffar4	1	+	TCGTGGAGACCACCGTTCTG	GGG
	2	-	CACGACGAGCACTAGAGGGA	TGG

Table S6: The sequence of primers used in the generation of mouse ileal knockout organoids.

Gene	Primers	Tm	WT band size	KO band size
Ffar1	F-GTGAACAGGAGGAACAGTGG	58 ℃	446 bp	403 bp
	R-AGGACAAGGGCCCATATAGC			
Gpr119	F-GAGAATCTGAGCTCGCCATC	58 ℃	519 bp	294 bp
	R-GGTCCATGGTAGGTGGTCTG			
Ffar4	F-CAAGTCAATCGCACCCACTT	58 ℃	589 bp	450 bp
	R-CGGCTTTGGTCAGATCCTTG			

Table S7: The sequence of oligos used in synthesis of sgRNA plasmid for generating human ileal KO organoids.

Targeting gene	Strand	gRNA sequence	PAM	Oligos	
FFAR1	+	CTGGTCTACGCCCTGAACCT	GGG	Forward	caccgCTGGTCTACGCCCTGAACCT
				Reverse	aaacAGGTTCAGGGCGTAGACCAGc
	-	CGGAAGGCTTGGTAGCCCAA	GGG	Forward	caccgCGGAAGGCTTGGTAGCCCAA
				Reverse	aaacTTGGGCTACCAAGCCTTCCGc
GPR119	+	AGAGATGGCCACACCAATCA	AGG	Forward	caccgAGAGATGGCCACACCAATCA
				Reverse	aaacTGATTGGTGTGGCCATCTCTc
	+	AAGTGACAAATGCCATCCGC	AGG	Forward	caccgAAGTGACAAATGCCATCCGC
				Reverse	aaacGCGGATGGCATTTGTCACTTc

Table S8: The sequence of primers used in the generation of human ileal knockout organoids.

Gene	Primers	Tm	WT band size	KO band size
FFAR1	F-AGCCAGGTTGCACACAGGAG	59.1 ℃	540 bp	344 bp
	R-GTTGACCGGTGTGTTGATGC			
GPR119	F-AGTGAAGTCCTGCCACTTCG	58 ℃	424 bp	352 bp
	R-AAGACACTAACCACAGCCCG			

Table S9: Physicochemical characterization of concentrated lipid-based nanocarriers and lipid nanocapsules replaced functional excipients (mean ± SEM, n=3).

Formulations	Size (nm)	PDI	Zeta potential
Concentrated 30 nm RM LNC	35.27±0.32	0.069±0.01	-0.96±0.06
Concentrated 200 nm RM LNC	200.2±2.40	0.071±0.02	-3.97±0.30
30 nm LNC (Lin/Pec)	38.23±0.99	0.267±0.01	-6.97±2.05
200 nm LNC (Lin/Pec)	197.4±0.28	0.176±0.01	-21.2±0.451
